# BI-D1870 Induces Mitotic Dysfunction and Apoptosis in Neuroblastoma by Regulating the PI3K-Akt-mTORC1 Signal Axis

**DOI:** 10.3390/cancers15072023

**Published:** 2023-03-28

**Authors:** Liming Jin, Tao Mi, Xin Wu, Zhang Wang, Zhaoxia Zhang, Jiayan Liu, Zhaoying Wang, Jinkui Wang, Mujie Li, Chunnian Ren, Peng Guo, Dawei He

**Affiliations:** 1 Department of Urology, Children’s Hospital of Chongqing Medical University, Chongqing 400014, China; 2 Chongqing Key Laboratory of Children Urogenital Development and Tissue Engineering, Chongqing 400014, China; 3 China International Science and Technology Cooperation base of Child Development and Critical Disorders, National Clinical Research Center for Child Health and Disorders, Ministry of Education Key Laboratory of Child Development and Disorders, Chongqing Key Laboratory of Pediatrics, Children’s Hospital of Chongqing Medical University, Chongqing 400014, China; 4 Institute of Basic Medicine and Cancer (IBMC), Chinese Academy of Sciences, Hangzhou 310022, China

**Keywords:** neuroblastoma, BI-D1870, RSK, mTORC1, G2/M arrest

## Abstract

**Simple Summary:**

To explore new drugs for malignant neuroblastoma, we used bioinformatics to analyze the prognostic genes, and successfully found the small molecular drug BI-D1870 based on the professional gene–drug analysis website ‘Connectivity Map’. In vivo and in vitro experiments confirmed that BI-D1870 effectively inhibited the malignant proliferation of neuroblastoma. Moreover, we found that BI-D1870 mainly inhibited the mitosis of tumor cells by regulating the PI3K-Akt-mTORC1 signal axis, thereby promoting cell apoptosis and ultimately inhibiting tumor growth. This is a drug prediction method based on a combination of massive gene map analysis and clinical survival practices, which has significant accuracy advantages over single gene changes. Furthermore, this is also the first study to explore the therapeutic benefits of BI-D1870 on NB, providing a novel clinically applicable solution for the treatment of neuroblastoma.

**Abstract:**

**Introduction:** Neuroblastoma (NB) is one of the most common extracranial solid malignant tumors in children. The 5-year survival rate of high-risk or refractory NB is less than 50%. Therefore, developing new effective therapeutics for NB remains an urgent challenge. **Materials and Methods:** Based on the NB dataset TARGET-NBL in the TCGA database, the prognosis-related genes were analyzed using univariate cox regression (*p* < 0.01). The protein network interaction of prognostic genes was analyzed using STRING to obtain 150 hub genes with HR > 1 and 150 hub genes with HR < 1. The Connectivity Map database was used to predict a therapeutic drug: BI-D1870, a ribosomal S6 kinase inhibitor. The inhibitory effect of BI-D1870 on NB was investigated through in vivo and in vitro experiments, and its inhibitory mechanism was explored. **Results:** Both the in vivo and in vitro experiments showed that BI-D1870 could inhibit tumor proliferation and induce tumor apoptosis. Furthermore, we proved that BI-D1870 caused G2/M phase arrest and mitosis damage in cells. RNA-seq of cells showed that BI-D1870 may inhibit the growth of NB by inhibiting the PI3K-Akt-mTOR axis. Western blot and immunofluorescence testing showed that BI-D1870 inhibited the PI3K-Akt-mTORC1 signal pathway to regulate the phosphorylation of RPS6 and 4E BP1 proteins, inhibit protein translation, and inhibit microtubule formation, thus preventing mitotic proliferation and inducing apoptosis. **Conclusions:** This study provides strong support that BI-D1870 may be a potential adjuvant therapy for NB.

## 1. Introduction

Neuroblastoma (NB) is one of the most common extracranial solid malignant tumors in children, accounting for 8–10% of pediatric tumors and about 15% of all childhood cancer deaths. In recent years, with the improvement in the social economy and healthcare, the diagnosis rate of NB in children has been continuously improved, and the prevalence rate has been increasing. It has become one of the most significant pediatric diseases affecting the health and life of children worldwide [1,2]. NB shows complex and diverse clinical manifestations based on its special biological characteristics. For the treatment of neuroblastoma, multidisciplinary comprehensive sequential therapy such as combined surgical treatment, chemical treatment, and radiotherapy is often used in the clinic. Nevertheless, the clinical outcome of neuroblastoma is still unsatisfactory, and there are serious problems such as chemotherapy insensitivity, multiple complications, and a high recurrence rate [3,4,5]. At present, the 5-year survival rate of neuroblastoma in developed countries with low/medium risk is 80% [1,6], while the overall survival rate of high-risk or refractory neuroblastoma is less than 50%. Finding new and effective measures for the treatment of neuroblastoma is a major challenge in current pediatric oncology research.

In recent years, with the intense research on neuroblastoma by clinicians and scientists, the treatment of NB in children has entered a new era of molecular biology. With the assistance of bioinformatic technologies, the current mainstream research focuses on identifying the molecular signatures of neuroblastoma based on multi-omics of genome and post-transcriptional modification, proteome, and post-translational modification [7,8,9]. Here, we used the prognosis-related genes of neuroblastoma to predict drugs based on the Connectivity Map database [1,10,11], and successfully identified the potential therapeutic drug BI-D1870 for neuroblastoma.

BI-D1870 is an ATP-competitive ribosomal S6 kinase (RSK) inhibitor that can specifically inhibit several subtypes of RSK proteins, including RSK1/RSK2/RSK3/RSK4 [12]. RSK is a highly conserved serine/threonine kinase that acts downstream of ERK signaling and mediates mitosis and stress-induced transcription factor activation [13,14]. It can regulate protein translation through phosphorylation of RPS6 and EIF4B and mediates cell proliferation, survival, and differentiation by regulating the mTOR signaling pathway [15,16]. Since malignant tumor formation is due to uncontrolled cell division and proliferation, and its occurrence and development are a multi-step continuous process, the role of protein kinase in the progression of malignant tumors is extremely critical [17]. Professor Iman Osman et al. reported that RSK1 activation is a feature of melanoma and is involved in its invasion and development [18]. Patrick Michl’s team proposed that RSK2 is activated in pancreatic cancer [19] and is a potential therapeutic target [20]. Professor Z Wang et al. reported that RSK4 could promote the cell cycle process, tumor invasion, and tumor migration of renal cancer cells, and may become a potential new therapeutic target for renal cancer patients [21]. BI-D1870 has been proven to have potent anti-tumor effects against a variety of malignancies, including melanoma, acute myeloid leukemia, lung cancer, and oral squamous cell carcinoma [22,23,24,25,26,27,28].

In this study, we explored the inhibitory effect of BI-D1870 on two neuroblastoma cell lines and studied the possible mechanism of BI-D1870 against NB cell activity.

## 2. Materials and Methods

### 2.1. NB Dataset

The clinical information and RNA-Seq data of NB were downloaded from the TCGA database using the TCGAbiolink package of R software (version 4.0.1). The screening criteria are as follows: (1) primary tumors and (2) samples with both mRNA expression data and clinical data. A total of 142 samples were obtained.

### 2.2. Prognostic Gene Screening and PPI Protein Network Analysis

Univariate Cox regression analysis was performed on all genes using the survival package in R software to obtain prognosis-associated genes. These genes were divided into prognosis-favorable (HR < 1) and prognosis-unfavorable groups (HR > 1). With the median expression value of each gene as the boundary, the genes were divided into high and low expression, and then a Kaplan–Meier survival curve was plotted (*p* < 0.01). In addition, the two groups of prognosis-related genes were subjected to protein–protein interaction networks (PPI) using the STRING database (https://cn.string-db.org/, accessed on 1 November 2021), and the top 150 hub genes in both groups were screened based on the degree values using Cytoscape software (version 3.8.0).

### 2.3. Drug Forecasting

The Connectivity Map (CMap) database (https://clue.io/, accessed on 23 November 2021) is a widely used drug prediction database that applies a genome-wide transcriptional profiling system to comprehensively describe the biological states of disease, physiology, drug induction, etc. The GSEA algorithm extracts and compares the gene expression signatures of these biological states, linking drugs of the same or opposite functions, drug-applicable diseases, and drug action pathways (genes, pathways). Here, we assume that if a drug can up-regulate the expression of genes with favorable prognosis and down-regulate the expression of genes with unfavorable prognosis, it may have therapeutic significance for this disease. We uploaded 150 hub genes of the favorable group and 150 hub genes of the unfavorable group to the CMap database and predicted a potential therapeutic agent for NB.

### 2.4. Structure of BI-D1870 and Its Target Protein RSK1

The drug structure schematic of BI-D1870 was obtained from the PubChem database (https://pubchem.ncbi.nlm.nih.gov/, accessed on 23 November 2021). The structural schematic of the target protein RSK1 was obtained from the UniProt database (https://www.uniprot.org/, accessed on 23 November 2021).

### 2.5. GO-KEGG Enrichment Analysis and GSEA Enrichment Analysis

The R software cluster profile package was used to analyze gene ontology (GO) and the Kyoto Encyclopedia of Genes and Genomes (KEGG) was used for genes with unfavorable prognoses. *p* < 0.05 was considered statistically significant. In addition, we performed gene set enrichment analysis (GSEA) on genes according to the HR value, and the gene set database was the Molecular Signatures Database (MSigDB, https://www.gsea-msigdb.org/gsea/msigdb/index.jsp, accessed on 27 November 2021).

### 2.6. Animal Experiments and Histopathology Experiments

#### 2.6.1. In Vivo Experiments

BALB/c nude mice were purchased from SPF Biotechnology (Beijing, China) and housed under specific pathogen-free conditions. The study was conducted according to international guidelines for the ethical treatment of animals and was approved by the Ethics Committee of the Children’s Hospital of Chongqing Medical University (Figure S1). One million SH-SY5Y cells were injected subcutaneously into the right axilla of 3-week-old male mice, and after the tumor volume reached 90–100 mm^3^, the mice were randomly divided into 4 groups with different doses (control, 10 mg/kg, 20 mg/kg, and 40 mg/kg) of BI-D1870 (MCE, 501437-28-1, Costa Mesa, CA, USA), with 6 mice in each group. The mice were injected intravenously every day for 7 days. The control group was injected with saline only. Body weight and tumor size were measured once a day (tumor volume = long × short^2^/2). At the end of the experiment, blood, subcutaneous tumor tissues, and major organs (heart, liver, kidney, lung, and spleen) were collected from the mice. One copy of each tissue was preserved in liquid nitrogen and 4% paraformaldehyde (Beyotime, P0099, Shanghai, China). Blood was collected from the orbit, and the serum was separated for liver function and kidney function tests, including ALT, AST, Cr, and BUN, by the Laboratory Department of the Children’s Hospital of Chongqing Medical University.

#### 2.6.2. HE Staining

The 4% PFA-fixed tissues were embedded in paraffin and 4 μm thick sections were prepared. Section samples were de-paraffinized and re-hydrated. Samples were treated with hematoxylin, eosin, a range of concentrations of alcohol, and xylene to complete HE staining. All images were observed and captured under an ECLIPSE Ci microscope (Nikon, Tokyo, Japan).

#### 2.6.3. Immunofluorescence Staining

For the immunofluorescence staining of the RSK1 protein, sections were de-paraffinized and subjected to antigenic thermal repair in citrate solution. Then, samples were treated with 3% hydrogen peroxide to block the endogenous peroxidase activity and blocked with 0.5% bovine albumin (A8020; Solarbio, Beijing, China). Anti-RSK1 antibody (ZENBIO, R2563, Chengdu, China) was incubated with sections for 12 h at 4 °C, followed by Cy3-conjugated secondary antibody (ZENBIO, 550076, China). Cell nuclei were stained using DAPI (Beyotime, C1006, China). All images were taken with an ECLIPSE 90i fluorescence microscope (Nikon, Japan).

#### 2.6.4. TUNEL

TUNEL (Beyotime, C1090, China) staining was performed to detect apoptosis of tumors according to the instructions. Samples were de-paraffinized and re-hydrated as described for fluorescent staining. Samples were then treated with proteinase K (20 μg/mL) and incubated with a TdT detection reagent. Cell nuclei were stained using DAPI. All images were taken with an ECLIPSE 90i fluorescence microscope.

### 2.7. Cell Lines and Cell Culture

NB cell lines SH-SY5Y, IMR-32, SK-N-SH, and SK-N-BE [2] were purchased from the Cell Collection of the Chinese Academy of Sciences (China) and SK-N-DZ was purchased from the American Type Culture Collection (ATCC, Manassas, VA, USA). All cells were cultured using high-glucose DMEM (MeilunBio, MA0212, Dalian, China) containing 10% fetal bovine serum (Corning, 35-076-CV, Corning, NY, USA) and incubated at 37 °C in an incubator containing 5% CO_2_.

### 2.8. Cell Proliferation Activity and Morphological Changes

The proliferative activity of the cells was measured using the CCK-8 assay (MCE, HY-K0301, USA). SH-SY5Y, SK-N-DZ, IMR-32, SK-N-SH, and SK-N-BE (2) cells were inoculated into 96-well plates and incubated completely for 12 h. The cells were replaced with a complete medium containing different concentrations of BI-D1870 and continued to be incubated for 48 h. The absorbance was measured at 450 nm after 2 h, and the half inhibitory concentration of BI-D1870 on cell proliferation was calculated. Then, SH-SY5Y and SK-N-DZ cells were intervened again using low, medium, and high concentrations (1/2 IC50, IC50, and 2 IC50) of BI-D1870, respectively, and cell CCK8 absorbance was measured after 24, 48, and 72 h of treatment to calculate the inhibition rate of proliferative activity. Meanwhile, the cell morphology after 48 h of BI-D1870 treatment was recorded.

### 2.9. Calcein/PI Cell Viability/Cytotoxicity Assay

Calcein/PI Cell Viability/Cytotoxicity Assay Kit (Beyotime, C2015M, China) was used to label BI-D1870-treated cells. SH-SY5Y and SK-N-DZ were inoculated into 24-well plates and incubated completely for 12 h. The cells were replaced with low, medium, and high concentrations of BI-D1870 complete medium and continued to be incubated for 48 h. The labeled cells were incubated with 1× Calcein AM and 1× PI for 2 h (37 °C, in dark). Images were all taken with an ECLIPSE 90i fluorescence microscope.

### 2.10. Cell Invasion Ability Test

Cell invasion ability was assayed using a wound healing assay and Transwell assay. SH-SY5Y and SK-N-DZ cells were inoculated into 6-well plates for culture and a wound was made on the fully fused cell surface. Cells continued to be cultured with serum-free medium containing a range of concentrations of BI-D1870, and the wound area was recorded by observing under an ECLIPSE microscope (Nikon, Japan) at 0, 24, and 48 h. SH-SY5Y and SK-N-DZ cells were inoculated into Transwell chambers (FALCON, 353097, Newport, TN, USA) of 24-well plates with matrix gel. The upper chamber of the 24-well plate contained serum-free medium, and the lower well contained complete medium, both containing a range of concentrations of BI-D1870. After 24 and 48 h of treatment, respectively, the chambers were removed and the cells were fixed with 4% paraformaldehyde and stained with a crystalline violet staining solution (Beyotime, C0121, China). Photographs were recorded under a light microscope and the number of cells was counted using ImageJ software (version 1.52).

### 2.11. Apoptosis through Flow Cytometry and TUNEL

Apoptosis was quantified using an Annexin V Apoptosis Kit (BD Pharmingen, 556547, Franklin Lakes, NJ, USA). SH-SY5Y and SK-N-DZ cells were completely cultured in 6-well plates for 12 h, changed to a medium containing different concentrations of BI-D1870, and continued to be cultured for 12, 48 (with or without 30 μM 740 Y-P, 740 Y-P was purchased from MCE, 1236188-16-1, USA) and 72 h. Annexin V detection reagent was added according to the instructions, and the percentage of apoptotic cells was detected using flow cytometry (BD Pharmingen, USA) and analyzed using FlowJo software (version 10.8.1). TUNEL staining was performed to detect apoptosis-induced DNA damage in cells from the medium- and high-dose intervention groups with significant apoptosis. SH-SY5Y and SK-N-DZ cells were inoculated into 24-well plate cell crawls and cultured completely for 12 h, changed to medium containing different concentrations of BI-D1870, and continued to be cultured for 48 h. TUNEL staining was performed according to the manufacturer’s instructions, and the nuclei were stained with DAPI and sealed by adding an anti-fluorescence quenching blocker dropwise. Photographs were recorded using a Nikon C2 laser confocal microscope (Nikon, Tokyo, Japan).

### 2.12. Cell Cycle Assay

Propidium iodide (PI)/RNase staining buffer solution (BD Pharmingen, 550825, USA) was used to detect the cell cycle. SH-SY5Y and SK-N-DZ cells were cultured completely in 6 cm dishes for 12 hr. The medium was changed to contain different concentrations of BI-D1870 and the culture was continued for12 or 48 hours (with or without 30 μM 740 Y-P). Cycle detection reagents were added according to the manufacturer’s instructions, and the cell cycle was detected using flow cytometry and analyzed with ModFit LT (version 3.2).

### 2.13. RNA Sequencing and Bioinformatics Analysis

Six samples of 0 or 1.28 μM BI-D1870-treated SH-SY5Y cells (three for each) were subjected to high throughput sequencing of mRNA. Total RNA was extracted from the cells using TRIzol^®^ reagent according to the manufacturer’s instructions (Invitrogen), and genomic DNA was removed using DNase I (Takara, Beijing, China). The RNA sample was used to construct a sequencing library. An RNA-seq transcriptome library was prepared following the TruSeqTM RNA sample preparation Kit from Illumina (San Diego, CA, USA) using 1 μg of total RNA. In short, messenger RNA was isolated according to the poly selection method using oligo(dT) beads, and then double-stranded cDNA was synthesized using a SuperScript double-stranded cDNA synthesis kit (Invitrogen, Carlsbad, CA, USA) with random hexamer primers (Illumina). Then, the synthesized cDNA was subjected to end-repair, phosphorylation, and ‘A’ base addition according to Illumina Libraries, where the size selected for cDNA target fragments was 300 bp on 2% low-range ultra agarose followed by PCR amplification using Phusion DNA polymerase (NEB) for 15 PCR cycles. After quantification using TBS380, the paired-end RNA-seq sequencing library was sequenced with an Illumina Hisses ten/NovaSeq 6000 sequencer (2 × 150 bp read length). DEGs are displayed using volcano maps and heat maps.

To identify DEGs (differential expression genes) between two different samples, the expression level of each transcript was calculated according to the RSEM (http://deweylab.biostat.wisc.edu/rsem/, accessed on 27 November 2021), which was used to quantify gene abundances. Essentially, differential expression analysis was performed using the DESeq2 with a *p* value < 0.05, and DEGs with |log2FC| ≥ 1 were considered to be significant. In addition, functional enrichment analysis including GO, KEGG, and GSEA was performed.

### 2.14. Cell Fluorescence Staining Experiment

Cell membrane staining was performed using a DIO Cell Membrane Staining Kit (Beyotime, C1993S, China), and F-Actin staining was performed using phalloidin (US EVERBRIGHT, YP0063, Hongkong, China). SH-SY5Y cells were completely cultured in 24-well plates for 12 h, changed to medium with different concentrations of BI-D1870, and continued to be cultured for 48 h. Cell membrane staining and phalloidin staining were performed according to the manufacturer’s instructions. Nuclei were stained using DAPI, and the number of nuclei within a single cell was recorded. SH-SY5Y cells were completely cultured in 24-well plates for 12 h, changed to a medium containing different concentrations of BI-D1870, and continued to be cultured for 48 h. Samples were sequentially fixed with 4% paraformaldehyde, closed with 0.5% BSA, incubated with β-Tubulin, CLIP170, and Aurora A primary antibodies, incubated with secondary antibodies, and nuclei-stained with DAPI. All images were recorded using a Nikon C2 laser confocal.

### 2.15. WB Experiment

SH-SY5Y cells were cultured in a medium containing different concentrations of BI-D1870 for 48 h. Cells were lysed using RIPA containing protease and phosphatase inhibitors and centrifuged at 4 °C (12,000× *g* for 20 min), supernatants were extracted, and protein quantification was performed using ta BCA kit. Then, 20 μg of protein was taken for SDS-PAGE gel electrophoresis (6–12.5% concentration, EpiZyme, Shanghai, China), with semi-dry electrotransfer of protein from SDS-PAG gel to PVDF membrane (Millipore, ISEQ00010, Burlington, MA, USA), fast closure solution for 10 min, primary antibody (1:1000) incubation for 12 h, and secondary antibody (1:5000) incubation for 1 h. Images obtained from the ChemiDoc MP Imaging System (Bio-Rad, Hercules, CA, USA) were analyzed in Image Lab (Version 3.0), showing protein blot (WB) images representing three independent replicates. The information of the antibodies is as follows: primary antibodies containing GAPDH (200306-7E4), RSK1 (R25632), PI3K (251221), p-PI3K (310164), Raptor (381155), RPS6 (R25622), p-RPS6 (310090), 4E BP1 (R24197), p-4E BP1 (R22929), CLIP170 (R26608), Aurora A (200525), and CDK1(200544) were purchased from ZENBIO (Chengdu, China). Primary antibodies containing Akt (60203-2-Ig), p-Akt (28731-1-AP), mTOR (66888-1-Ig), and p mTOR (67778-1-Ig) were purchased from Proteintech (Wuhan, China). Horseradish peroxidase-labeled secondary antibodies (ZB-5301 and ZB-2305) were purchased from ZSGB-BIO (Beijing, China).

### 2.16. Statistics

Data analysis was performed using GraphPad 8.0 software. Measurement data are expressed as mean ± standard deviation. One-way ANOVA was used to compare the means of three or four groups. The *t*-test was used to compare the means of two groups. *p* < 0.05 was considered statistically significant.

## 3. Results

### 3.1. Bioinformatics Analysis and Drug Prediction of Prognostis-Related Genes

**Figure 1:**
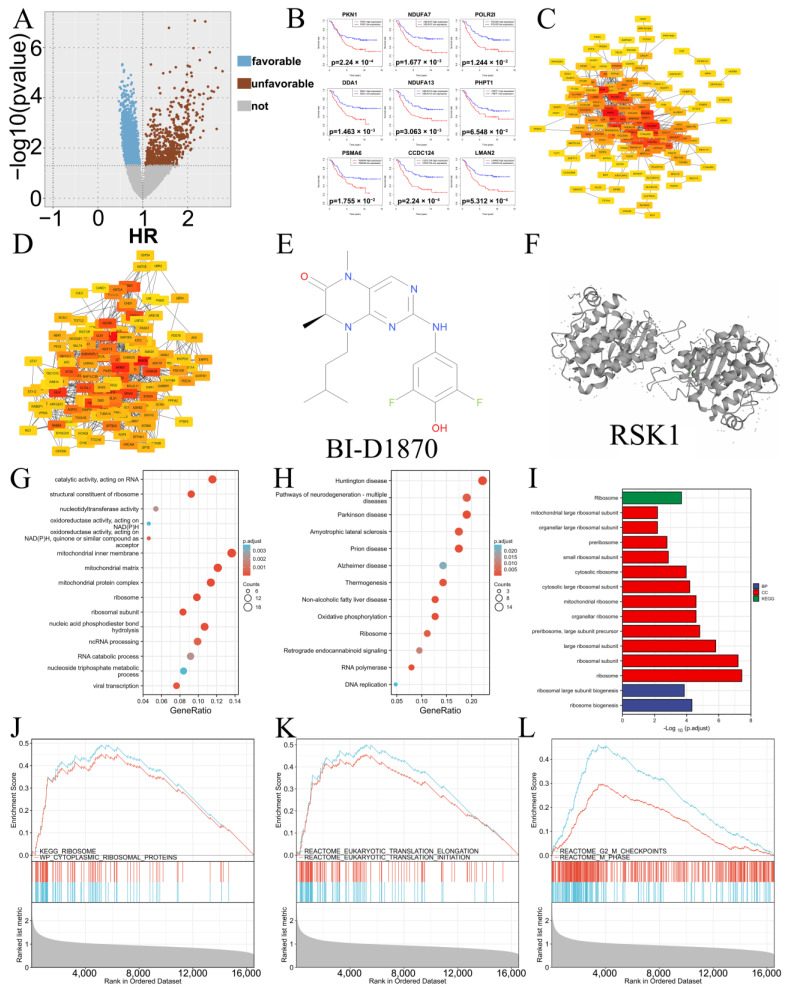
Bioinformatics analysis of prognosis-related genes and drug prediction in NB. (**A**) Volcano map of the prognosis-related gene. (**B**) The top nine unfavorable prognosis genes. (**C**) The 150 hub favorable prognosis genes. (**D**) The 150 hub unfavorable prognosis genes. (**E**) Structural formula of BI-D1870. (**F**) Structural formula of RSK1. (**G**) GO; (**H**) KEGG; (**I**) GO-KEGG of ribosome-associated pathways. (**J**) GSEA of ribosome-related pathways. (**K**) GSEA of protein translation-related pathways. (**L**) GSEA of G2/M phase-related pathways.

A total of 1178 prognosis-related genes were obtained through univariate Cox regression analysis (*p* < 1), and there were 919 favorable prognosis (HR < 1) and 259 unfavorable prognosis genes (HR > 1) (Figure 1A). Among them, the top nine unfavorable prognosis genes (with the highest HR values) were PKN1, NDUFA7, POLR2I, DDA1, NDUFA13, PHPT1, PSMA6, CCDC124, and LMAN2, in order (Figure 1B). Next, prognosis-related genes were analyzed through protein–protein interaction (PPI) network analysis. The first 150 hub genes (Figure 1C,D) of favorable and unfavorable genes were obtained from the STRING database. Based on two sets of hub genes, the Connectivity Map database (https://clue.io/, accessed on 23 November 2021) was used to predict a potential therapeutic drug for NB, an ATP-competitive ribosomal S6 kinase (RSK) inhibitor—BI-D1870 (Figure 1E,F). GO-KEGG enrichment analysis showed that 150 hub unfavorable prognosis genes were mainly enriched in ribosome composition and biological function, mitochondrial biological function, oxidative phosphorylation, DNA replication, nucleic acid metabolism, and other pathways (Figure 1G,H). Favorable prognosis genes were heavily enriched in ribosome composition and biological function-related pathways (Figure 1I). Meanwhile, all genes in the dataset were subjected to GSEA enrichment analysis based on HR values, and it was also found that they were enriched in the KEGG-RIBOSOME and WP-CYTOPLASMIC RIBOSOME PROTEIN signaling pathways (Figure 1J). It can be concluded that the unfavorable prognosis of NB is closely related to ribosomal biological function. In addition, GSEA enrichment analysis found that the eukaryotic translation signaling pathway and G2/M phase signaling pathway were also associated with an unfavorable prognosis of NB (Figure 1K,L).

### 3.2. In Vivo Inhibitory Effect

**Figure 2:**
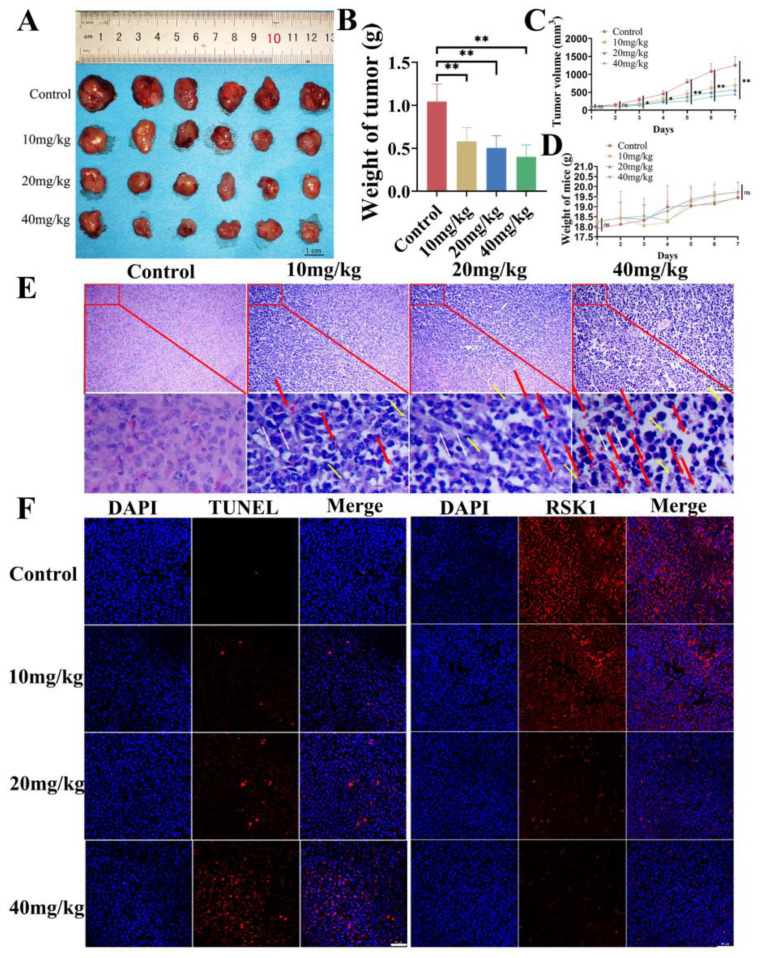
In vivo therapeutic effect of BI-D1870. (**A**) Tumor tissues. (**B**) Weights of tumors. (**C**) Tumor growth curve. (**D**) Body weight of mice. (**E**) HE staining of tumors, scale bar: 100 μm; white arrow: intercellular cavities; yellow arrow: cell necrosis; red arrow: localized hemorrhage. (**F**) TUNEL, scale bar: 100 μm. Immunofluorescence of RSK1, scale bar: 100 μm. ns *p* > 0.05, * *p* < 0.05, ** *p* < 0.01.

NB tumor-bearing animals were treated with BI-D1870 with three different dosages (10, 20, 40 mg/kg) in order to determine its in vivo inhibitory effect. The subcutaneous tumor volume was monitored continuously, and the tumor volumes of all three BI-D1870 treated groups were found to be significantly smaller than that of the control group for the first time since day 3, and the inhibitory effect was improved with increasing BI-D1870 dosages. BI-D1870 administrated at 40 mg/kg mediated the most potent in vivo inhibitory effect, shrinking the NB tumor weight by 60% in comparison with the PBS group (Figure 2A–D). HE staining indicated that the tumor cell cytoplasmic proteins were significantly reduced after BI-D1870 treatment, and the tumor cells showed intercellular cavities, localized hemorrhage, and cell necrosis (Figure 2E). The RSK1 protein was the target of BI-D1870, and the immunofluorescence results demonstrated the efficacy of drug action in the tumors of the intervention groups (Figure 2F). HE staining of the heart, liver, kidney, lung, and spleen suggested no significant differences between the groups. The groups did not statistically differ between liver and kidney function (Figure S2).

### 3.3. Cytotoxicity of BI-D1870

**Figure 3:**
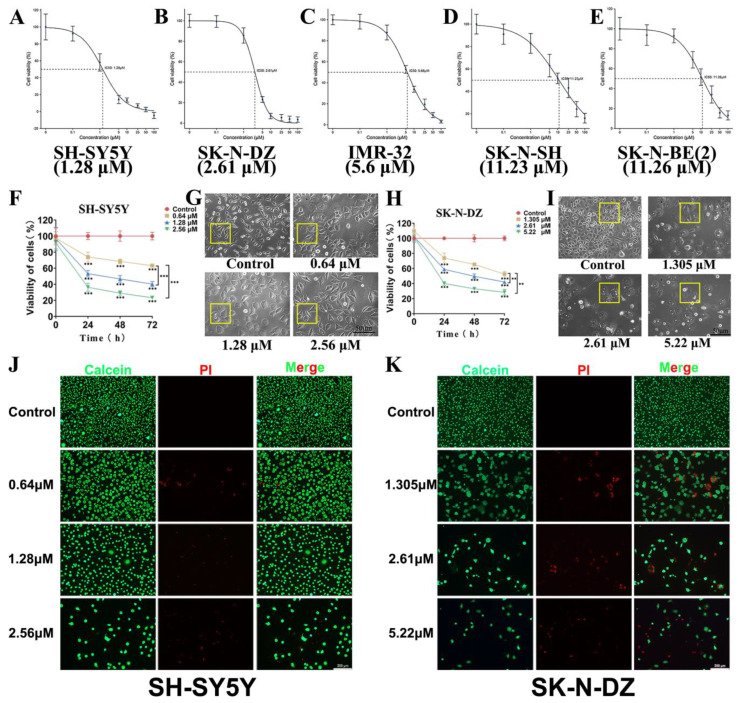
Cytotoxicity of BI-D1870. (**A**–**E**) IC50 of BI-D1870 to SH-SY5Y, SK-N-DZ, IMR-32, SK-N-SH, and SK-N-BE (2). (**F**) Proliferation curve of SH-SY5Y. (**G**) Morphology of SH-SY5Y, scale bar: 50 μm. (**H**) Proliferation curve of SK-N-DZ. (**I**) Morphology of SK-N-DZ, scale bar: 50 μm. (**J**,**K**) dead/living cell staining of SH-SY5Y and SK-N-DZ, scale bar: 200 μm.** *p* < 0.01, *** *p* < 0.001.

The CCK8 assay was used to determine the proliferation inhibitory effect of BI-D1870 on NB cell lines. The IC50 values for SH-SY5Y, SK-N-DZ, IMR-32, SK-N-SH, and SK-N-BE (2) were determined to be 1.28 μM, 2.61 μM, 5.6 μM, 11.23 μM, and 11.26 μM (Figure 3A–E), respectively, after 48 h of BI-D1870 treatment of the cells. Next, the highly sensitive SH-SY5Y and SK-N-DZ cells were treated with low, medium, and high doses (1/2 IC50, IC50, and 2 IC50) of BI-D1870, and we found that cells in the treated group showed a dose-dependent decrease in proliferative activity compared to the control group and significant changes in cell morphology. BI-D1870-treated cells clearly lost their filopodia architecture and became more cobblestone-shaped with dense and visible intracellular granules (Figure 3F–I). The dead/live cell staining suggested that some dead cells appeared in the BI-D1870-treated groups, and the proportion of dead cells increased significantly in the high-dose group (Figure 3J,K).

### 3.4. BI-D1870 Inhibited Cell Migration and Invasion Ability

**Figure 4:**
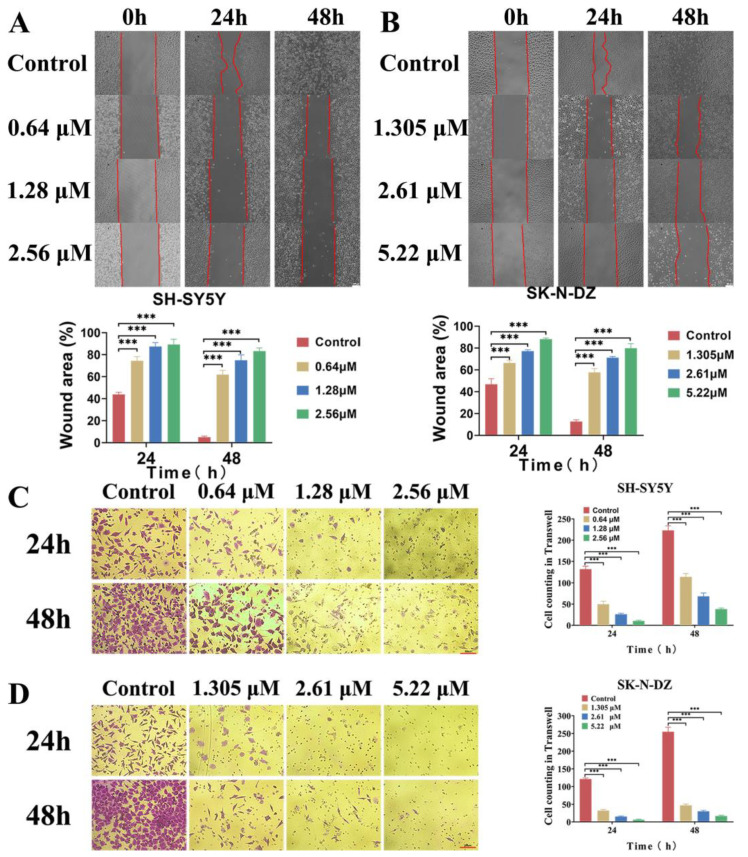
BI-D1870 inhibits migration and invasion. (**A**,**B**) Wound healing assay of SH-SY5Y and SK-N-DZ, scale bar: 100 μm. (**C**,**D**) Transwell assay of SH-SY5Y and SK-N-DZ. *** *p* < 0.001.

The wound healing assay suggested that the cell migration rates of SH-SYY5Y and SK-N-DZ cells in the BI-D1870-treated group were significantly slower than the control group at 24 h and 48 h post-treatment (Figure 4A,B). After 24 and 48 h of BI-D1870 treatment, the Transwell assay also showed that the number of cells crossing the matrigel was significantly reduced, indicating that BI-D1870 could reduce the cell invasion capability of NB cells (Figure 4C,D).

### 3.5. BI-D1870-Induced Cell Apoptosis

**Figure 5:**
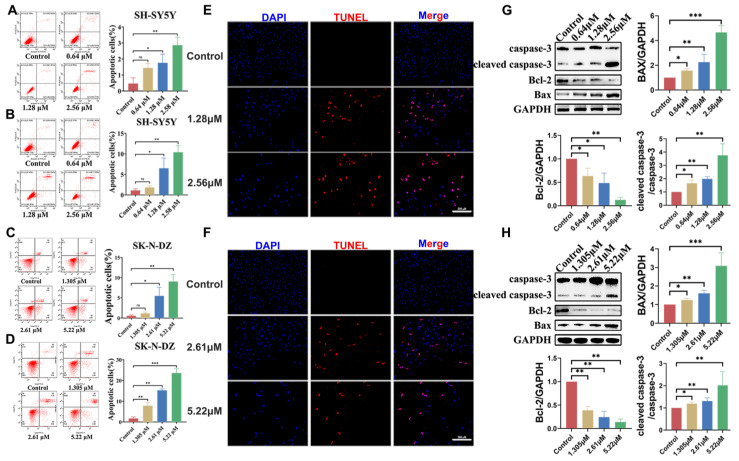
BI-D1870 induces apoptosis. (**A**–**D**) Annexin V apoptosis assay in SH-SY5Y and SK-N-D. (**E**,**F**) TUNEL of SH-SY5Y and SK-N-D, scale bar: 200 μm. (**G**,**H**) Apoptosis-related proteins in SH-SY5Y and SK-N-D. ns *p* > 0.05, * *p* < 0.05, ** *p* < 0.01, *** *p* < 0.001. The uncropped blots and molecular weight markers are shown in Supplementary File.

The Annexin-V apoptosis assay revealed that significantly increased levels of apoptosis occurred in treated cells, which increased with treatment time. The apoptotic response of the two cell lines was inconsistent, with SK-N-DZ showing a more pronounced apoptosis sensitivity. The percentage of apoptotic cells in the SK-N-DZ group increased from less than 1% in the control to approximately 10% after 48 h of treatment at high doses, and increased to 25% when the treatment time was extended to 72 h. The same phenomenon was echoed in the SH-SY5Y cells, where only 3% of the cells showed apoptosis at 48 h post-treatment, while the percentage of apoptotic cells increased threefold at 72 h (Figure 5A–D). We performed TUNEL assays on two cell lines in the medium- and high-dose groups and found positive TUNEL assays for apoptotic cells in the treated group, implying that a late apoptotic DNA damage response occurred after treatment (Figure 5E,F). WB experiments revealed that after 48 h of BI-D1870 treatment, the apoptotic execution protein in both cell lines’ cleaved caspase-3 expression was significantly up-regulated and the expression of the pro-apoptotic protein Bax was also significantly increased, while the expression of the apoptosis inhibitory protein Bcl-2 was significantly suppressed in both cell lines after 48 h of BI-D1870 treatment (Figure 5G,H). It was verified at the protein level that BI-D1870 treatment can increase NB cell apoptosis.

### 3.6. BI-D1870 Induces G2/M Arrest and Mitotic Catastrophe

**Figure 6:**
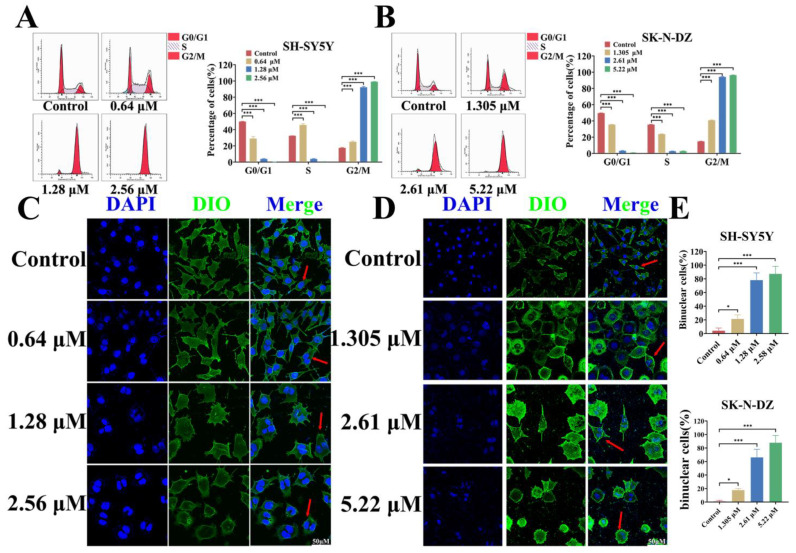
BI-D1870 induces G2/M phase block and multinucleated cell formation. (**A**,**B**) The cell cycle of SH-SY5Y and SK-N-DZ. (**C**,**D**) Cell membrane fluorescence staining, scale bar: 50 μm. red arrows: Binuclear cells. (**E**) The number of nuclei. * *p* < 0.05; *** *p* < 0.001.

Flow cytometric cycle experiments showed a significant increase in the proportion of cells in the G2/M phase in both NB cell lines after BI-D1870 treatment, especially in the medium- and high-dose groups (over 90%) (Figure 6A,B). We further performed cell membrane and nucleus fluorescence staining on the treated cells, and observed that two nuclei appeared in a large number of cells (Figure 6C,D). After treatment with BI-D1870, the proportion of binuclear cells significantly increased (Figure 6E). This suggests that BI-D1870 may inhibit the normal cell cycle and prevent the regular progress of mitosis, resulting in impaired cell division.

### 3.7. PI3K-Akt-mTORC1 Signaling Pathway Inhibition

**Figure 7:**
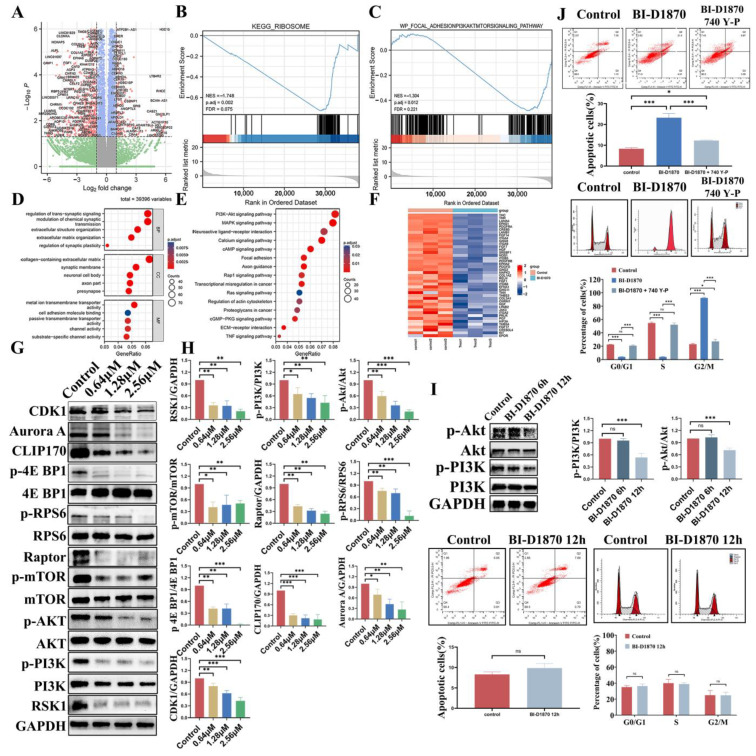
RNA-seq, bioinformatics analysis, and PI3K-Akt-mTORC1 signaling pathway detection of SH-SY5Y. (**A**) Volcano map. (**B**,**C**) GSEA of DEGs. (**D**,**E**) GO-KEGG. (**F**) Heat map of PI3K-Akt-mTOR. (**G**,**H**) WB. (**I**) 0, 6, and 12 h after BI-D1870 treatment. (**J**) 740 Y-P and BI-D1870 coprocessing. ns *p* > 0.05; * *p* < 0.05, ** *p* < 0.01, *** *p* < 0.001. The uncropped blots and molecular weight markers are shown in Supplementary File.

RNA-seq of SH-SY5Y cells treated for 48 h revealed that BI-D1870 down-regulated the expression of 1243 genes and up-regulated the expression of 487 genes (Figure 7A).

GSEA analysis suggested that the “RIBOSOME” and “PI3K-Akt-mTOR” signaling pathways were significantly inhibited (Figure 7B,C). GO and KEGG analysis identified “PI3K-Akt” as the pathway with the highest enrichment factor signal (Figure 7D,E). A large number of differential genes in the treated and control groups were enriched in this pathway (Figure 7F). This suggests that BI-D1870 is likely to inhibit NB growth by regulating the PI3K-Akt-mTOR signaling pathway.

We used Western blotting to detect the changes in protein levels. After 48 h treatment with BI-D1870 for SH-SY5Y, it was found that the expression of the RSK1 protein was significantly decreased, which confirmed that the intervention was effective. It is known that RSK participates in the mTOR signaling pathway to regulate protein biosynthesis and microtubule formation. We found that BI-D187 significantly down-regulated the phosphorylation levels of the PI3K, Akt, and mTOR proteins. Additionally, the expression of Raptor, the key protein of the mTORC1 complex, was significantly decreased. In addition, the phosphorylation of the mTOR downstream proteins RPS6 and 4EBP1 also decreased significantly. This suggests that BI-D1870 can inhibit cell protein synthesis by inhibiting PI3K-Akt-mTORC1 pathway activity, resulting in decreased ribosomal biological function and the inhibition of protein translation. Meanwhile, the mTORC1 downstream key protein CLIP170 is involved in the regulation of microtubule formation and stability. We found that the expression of the CLIP170 protein in the treated cells was also significantly down-regulated. The Aurora A protein involved in centrosome separation and spindle poles was also down-regulated in the treated cells. The activation of the cycling-CDK1 complex was related to the cell cycle checkpoint at the G2/M phase, and the expression of the CDK1 protein decreased significantly after BI-D1870 treatment (Figure 7G,H). In addition, we found that protein phosphorylation of the PI3K-AKT signaling pathway was significantly inhibited when cells were treated with BI-D1870 for 12 h. However, at this time, we did not observe significant cell cycle arrest or increased apoptosis (Figure 7I). Then, NB cells were treated with BI-D1870 in combination with 740 Y-P (an AKT agonist), and we found that the proportion of apoptosis after adding 740 Y-P was significantly lower than using BI-D1870 alone, and the cell cycle arrest was also significantly alleviated (Figure 7J).

### 3.8. BI-D1870 Induces Disruption of Cytoskeleton Structure

**Figure 8:**
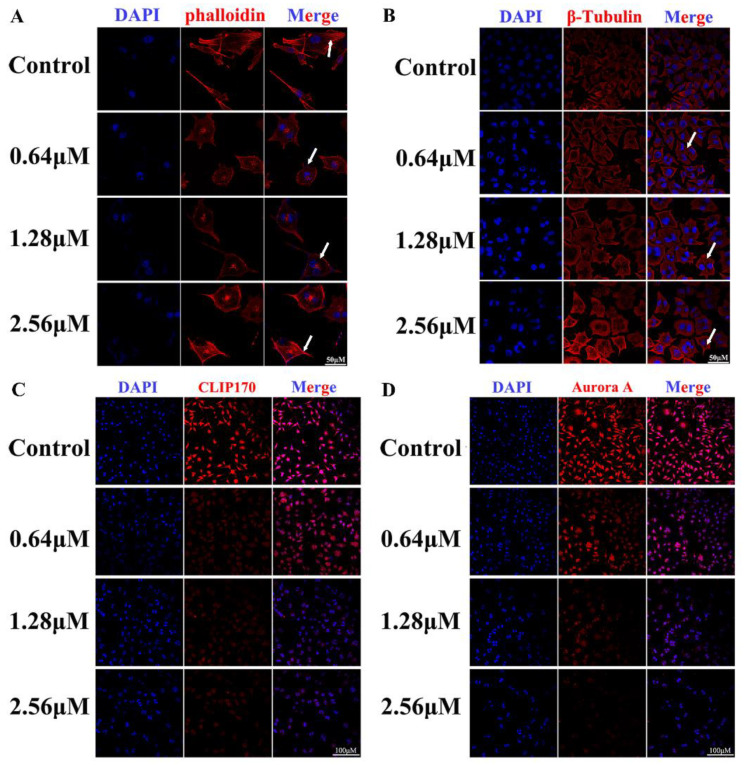
BI-D1870 induced dysregulation of cellular microtubule structure in SH-SY5Y. (**A**) Phalloidin staining, scale bar: 50 μm. (**B**–**D**) β-Tubulin, scale bar: 50 μm, CLIP170, and Aurora A staining of SH-SY5Y, scale bar: 100 μm.

To further investigate how BI-D1870 regulates mitosis, we investigated the cytoskeleton structure and skeleton regulatory proteins in SH-SY5Y. Phalloidin staining revealed that F-actin was uniformly distributed and the microfilament skeleton was spreading in the control cells, while the actin fibers were disordered and the cytoskeleton was remodeled after BI-D1870 treatment (Figure 8A). Cellular immunofluorescence β-Tubulin staining revealed that the control cells had clear microtubule distribution and cytoskeleton structure, while treated cells exhibited intracellular multinuclear aggregation, wide gaps between microtubes, and dysregulated microtubule skeleton (Figure 8B). In addition, we detected that the levels of the CLIP170 and Aurora A proteins, which regulate microtubule formation and stabilization, were also significantly decreased (Figure 8C,D).

## 4. Discussion

Based on the clinical information and RNA-Seq data of NB patients in the TARGET database, the genes related to the prognosis of NB were analyzed. Based on the protein network interaction relationship, 150 hub genes were screened in the favorable prognosis group and 150 hub genes in the unfavorable prognosis group. Then, we predicted that BI-D1870 could be used as a potential therapeutic drug for NB through the CMap drug database. Subsequently, we performed GSEA analysis of all prognosis-related genes and GO-KEGG functional enrichment analysis of unfavorable prognosis group hub genes and found that a large number of unfavorable prognosis genes were enriched in pathways related to ribosomal biological functions, protein synthesis, and the cell cycle. This is consistent with the biological characteristics of malignant proliferation and high protein synthesis of NB.

The target protein of BI-D1870 is the ribosomal protein S6 kinase, a member of the serine/threonine kinase family. It mediates mitosis and transcription factor activation through the ERK signaling pathway and regulates protein translation, cell proliferation, and survival through the mTOR signaling pathway. The PI3K/mTOR signaling pathway has been reported to be persistently active in NB, leading to dysregulation of the cellular metabolism [29,30,31]. Moreover, high PI3K/mTOR activity and phosphorylated Akt are associated with resistance and an unfavorable prognosis of NB [32].

In this study, we found that BI-D1870 significantly inhibited the survival of NB cells by inducing impaired mitosis and activating apoptosis. This study indicates that the proliferation inhibition of NB cells by BI-D1870 appears to be independent of whether the cells themselves are accompanied by MYCN gene amplification mutations, implying that it is likely that BI-D1870 can address the therapeutic challenge of high-risk NB with MYCN gene amplification, which is insensitive to conventional tumor chemotherapeutic agents. Marcus et al. reported that BI-D1870 exerted an anti-melanoma effect by inducing apoptosis [25]. Ayane et al. reported that BI-D1870 can induce apoptosis of acute myeloid leukemia cells [26]. We also examined the apoptosis of NB cells after BI-D1870 treatment and found that the percentage of apoptotic cells did increase, but the extent of the increase was much lower than we expected after 48 h of treatment. Only about 3% of the high-dose BI-D1870-treated SH-SY5Y cells underwent apoptosis. In SK-N-DZ cells, where apoptosis was more pronounced, only about 10% of the cells underwent apoptosis, even though the level of apoptosis increased nearly 10-fold after treatment compared to the control group. The apoptotic results could not explain such a pronounced inhibitory effect of BI-D1870 on NB cells as we observed. However, when the treatment time was extended to 72 h, we were surprised to find a tremendous increase in the apoptosis level of both cells, with a 10-fold increase in the percentage of apoptosis in SH-SY5Y cells compared to control cells, and a 20-fold increase in SK-N-DZ cells. This suggests that BI-D1870-induced apoptosis of tumor cells takes longer and the drug efficacy increases with treatment time.

Bioinformatics analysis of NB unfavorable prognosis genes suggests their substantial enrichment in cell cycle-related pathways. Based on the theory that RSK family proteins are involved in mTOR and ERK signaling pathway-mediated protein translation and mitosis-mediated cell proliferation, we investigated the effect of BI-D1870 on the cell cycle. Fatim et al. reported that BI-D1870 induced G2/M phase arrest and inhibited lung cancer cell proliferation [24]. Weng et al. also reported that BI-D1870 induced G2/M phase arrest of human oral squamous cell carcinoma [22]. In this study, as expected, both NB cell lines showed significant G2/M arrest after treatment. Moreover, we labeled the cell membrane and nucleus of SH-SY5Y cells with fluorescent dyes, and we found that a large number of cells had double nuclei, which further verified our hypothesis that the treated cells had a mitotic disorder. In addition, apoptosis and cell cycle arrest were significantly alleviated after the use of Akt agonists, which further supports our reasoning.

To investigate how BI-D1870 inhibits cell survival, we performed RNA sequencing and transcriptomic analysis on treated SH-SY5Y cells. DEGs analysis identified 1243 genes with down-regulated expression and 487 genes with up-regulated expression. Further KEGG and GSEA enrichment analysis based on transcriptomic data showed that the most enriched pathway was “PI3K-Akt-mTOR”, which was significantly repressed in the treated group. This reasonably explains our previous findings; that is, the cells in the treated group showed significant cell cycle arrest, mitotic catastrophe, proliferation inhibition, and apoptosis activation. Based on the PI3K-Akt-mTOR signaling pathway, we performed subsequent studies on the regulation of proteins centered on the mTORC1 complex. Other important enrichment pathways include the “MAPK signaling pathway, Calcium signaling pathway, Transcriptional misregulation in cancer, regulation of actin cytoskeleton, ECM-receptor interaction”, which will be further explored in our subsequent studies.

RSK can regulate the mTORC1 complex in the mTOR signaling pathway, and then later participate in cell protein translation and microtubule formation and stability [33,34]. The progress of mitosis in mammalian cells depends on protein synthesis before division and spindle-mediated cytoplasmic division during division [35,36,37]. In this study, we found that after using BI-D1870 to inhibit RSK, the phosphorylation activity of the mTORC1 complex was significantly inhibited, and the expression of its main component, Raptor [38], was significantly decreased. mTORC1 regulates the expression and phosphorylation of the eukaryotic translation initiation factor eIF4B protein by regulating the phosphorylation of the downstream proteins RPS6 and 4EBP1, thereby regulating the protein translation process [19,39,40]. The low-phosphorylated 4E BP1 protein strongly binds to eIF4 B and inhibits translation initiation, while the over-phosphorylated 4E BP1 protein relieved the inhibition of eIF4 B. The eIF4 B mediates mRNA and ribosome binding to initiate protein translation [41,42]. The phosphorylated RSK6 protein continues phosphorylating the downstream eIF4B protein to initiate protein translation [43]. In this study, we observed that after BI-D1870 treatment, 4E BP1 showed a low phosphorylation level, and RSK6 phosphorylation was significantly decreased. Both of them inhibited eIF4 B to inhibit protein translation [44,45,46]. CLIP1, a protein in downstream mTORC1, binds to the positive end of microtubules and regulates the dynamics of the microtubule cytoskeleton to promote the growth and connection of microtubules. We found the expression of CLIP170 protein decreased after treatment. During mitosis, the Aurora A protein connects centrosomes and spindle microtubules and plays a key role in a variety of mitosis processes, including the formation of mitotic spindles, centrosome replication, isolation and maturation, chromosome arrangement, spindle assembly checkpoint, cytoplasmic division, and the initial activation of CDK1 at centrosomes [47,48,49]. Similarly, we found a decrease in Aurora A protein expression in treated cells. In addition, fluorescence staining of the cytoskeleton revealed that both F-Actin and β-Tubulin showed structural disorganization and remodeling in the treated groups. It was confirmed that BI-D1870 inhibited mTORC1-regulated microtubule stability by inhibiting RSK.

At present, the application research of BI-D1870 in tumor treatment is still in its infancy. There are only a small number of studies that have noted the possible anti-tumor efficacy of BI-D1870. Dr. Abdulrahman found that BI-D1870 can mitigate tumor growth and potentiate cisplatin activity in LUAD cells through phospho-GSK-3β and osteopontin [23,24]. Dr. Nada reports that BI-D1870 enhances the killing effect of erlotinib on pancreatic cancer cells by inhibiting RSK3 targets [20]. Dr. Immacolata found that BI-D1870 induces apoptosis and proliferation arrest of thyroid cancer cells by reducing MDM2 phosphorylation and restoring P53 function [50]. These studies suggest that BI-D1870 may be an effective new attempt in cancer treatment, with many opportunities to be discovered. At the same time, we must point out that all current research on the treatment of tumors with BI-D1870 only focuses on tumor cell experiments and mouse experiments. As yet, we have not seen any clinical studies on BI-D1870. Whether BI-D8170 can be truly converted into clinical drugs still requires continuous exploration through follow-up research. Currently, there are still many issues waiting to be resolved, such as pharmacokinetics, targeted modification, human safety dose and effective dose, and so on.

## 5. Conclusions

In summary, we analyzed the prognosis-related genes of NB using bioinformatics technology, identified a potential therapeutic drug, BI-D1870, and confirmed that BI-D1870 can effectively inhibit the growth of NB. We found that BI-D1870 inhibited the phosphorylation of downstream RPS6 and 4E BP1 by inhibiting the PI3K-Akt-mTORC1 signaling axis, and inhibited protein translation initiation. BI-D1870 also inhibited mTORC1-mediated microtubule formation and stability. Both of these induce cell G2/M phase arrest and mitotic disorder, and ultimately inhibit NB. Meanwhile, damaged tumor cells stimulate apoptosis in order to be self-cleaned, demonstrating the tumor-killing effect of BI-D1870. Overall, the evidence of this study provides strong support for the further exploration of BI-D1870 or other RSK inhibitors as potential adjuvant therapy for NB.

## Data Availability

The NB datasets generated and analyzed during the current study are available in the TCGA repository [https://portal.gdc.cancer.gov/projects/TARGET-NBL] (accessed on 1 November 2021). The RNA-seq results of the SH-SY5Y cells have been uploaded to the Sequence Read Archive (submission: SUB12282507).

## Supplementary Materials

The following supporting information can be downloaded at: https://www.mdpi.com/article/10.3390/cancers15072023/s1, Figure S1: HE staining, liver function, and renal function. (A) HE Staining of heart, liver, kinder, lung and spleen, Scale bar: 100 μm; (B) ALT, AST, Cr and BUN, ns *p* > 0.05; Supplementary File: The uncropped blots and molecular weight markers for Figure 5G,H and Figure 7G,I.
