# Supplementary material for: BI-D1870 Induces Mitotic Dysfunction and Apoptosis in Neuroblastoma by Regulating the PI3K-Akt-mTORC1 Signal Axis
Source: Cancers (Basel). 2023 Mar 28;15(7):2023. doi: 10.3390/cancers15072023 (PMC10093276; doi:10.3390/cancers15072023)
Supplement: Supplementary file 1 [file cancers-15-02023-s001.zip › cancers-2272070-Figure S1.pdf]

Supplementary Materials: BI-D1870 Induces Mitotic Catastrophe And Apoptosis In Neuroblastoma By Regulating PI3K-Akt-mTORC1

Liming Jin, Tao Mi, Xin Wu, Zhang Wang, Zhaoxia Zhang, Jiayan Liu, Zhaoying Wang, Jinkui Wang, Mujie Li, Chunnian Ren, Peng Guo and Dawei He

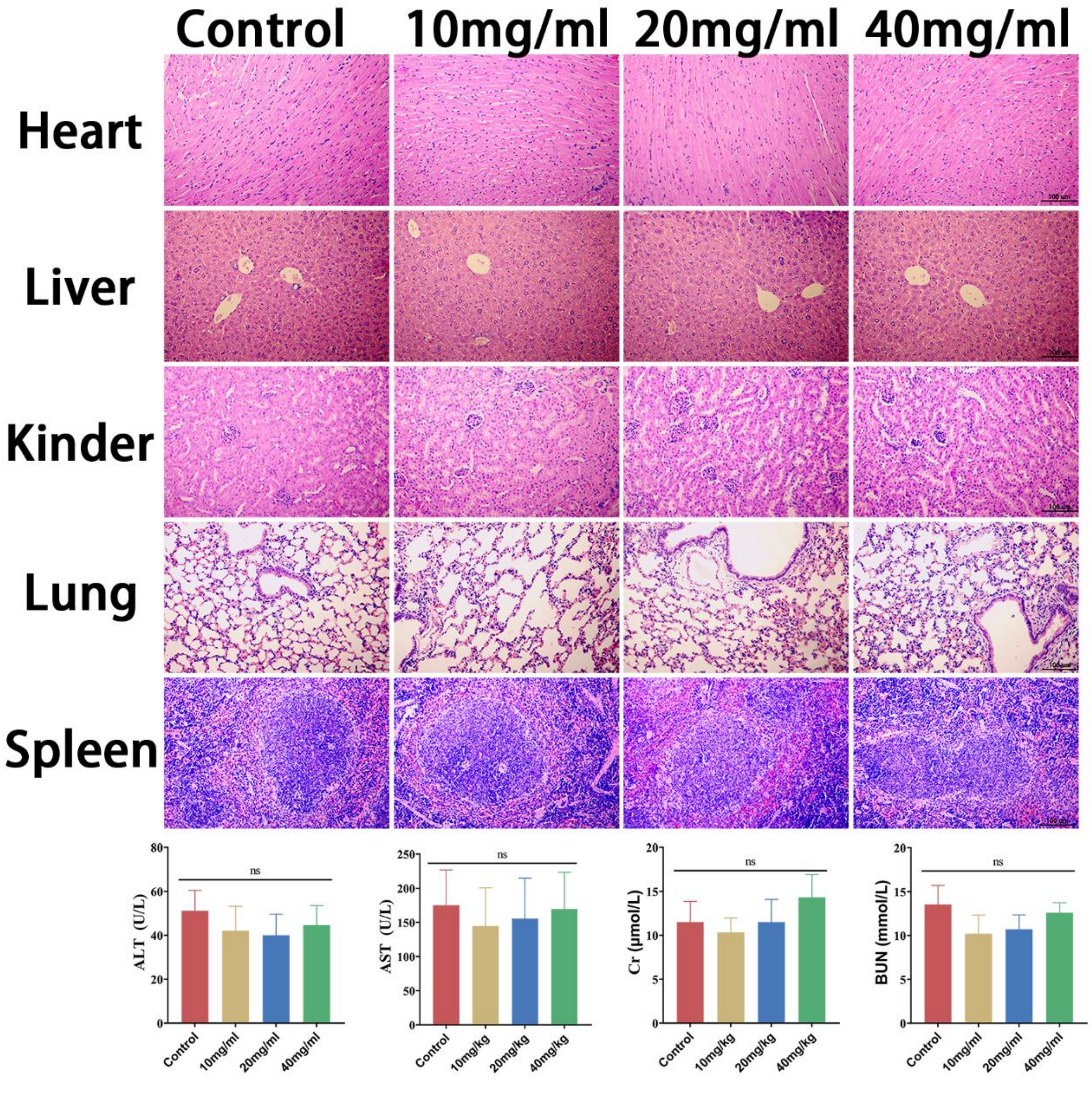

Figure S1. HE staining, liver function, and renal function.(A) HE Staining of heart, liver, kinder, lung and spleen, Scale bar: 100μm; (B) ALT, AST, Cr and BUN, ns $p > 0.05$.
