# Supplementary material for: BI-D1870 Induces Mitotic Dysfunction and Apoptosis in Neuroblastoma by Regulating the PI3K-Akt-mTORC1 Signal Axis
Source: Cancers (Basel). 2023 Mar 28;15(7):2023. doi: 10.3390/cancers15072023 (PMC10093276; doi:10.3390/cancers15072023)
Supplement: Supplementary file 1 [file cancers-15-02023-s001.zip › cancers-2272070-file.pdf]

GADPH

SH-SY5Y Cell

Control | 0.64μM BI-D1870 | 1.28μM BI-D1870 | 2.56μM BI-D1870 ; Control | 0.64μM BI-D1870 | 1.28μM BI-D1870 | 2.56μM BI-D1870

KDa

36

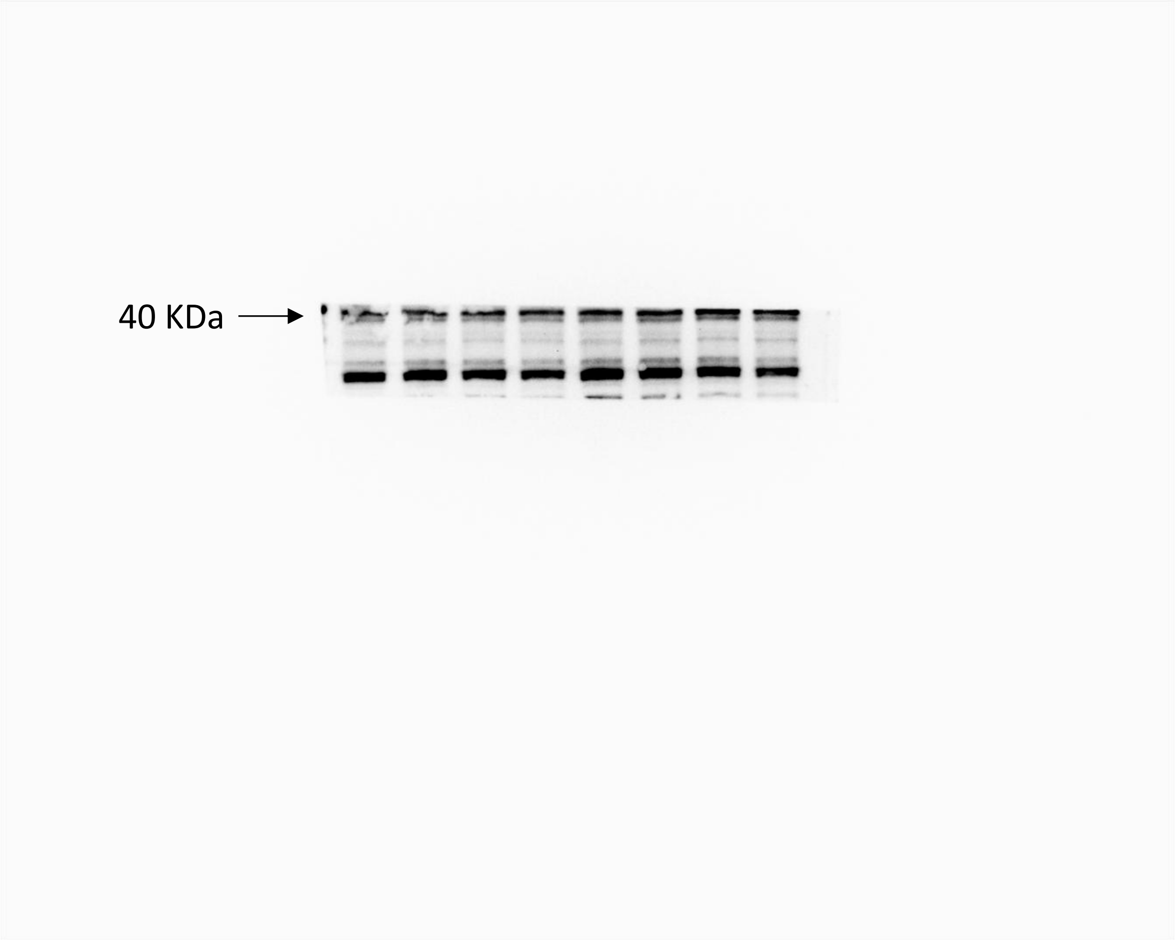

100 : 100 : 100 : 100 ; 100 : 100 : 100 : 100

Figure 5-G

Bax

SH-SY5Y Cell

Control | 0.64μM BI-D1870 | 1.28μM BI-D1870 | 2.56μM BI-D1870 ; Control | 0.64μM BI-D1870 | 1.28μM BI-D1870 | 2.56μM BI-D1870

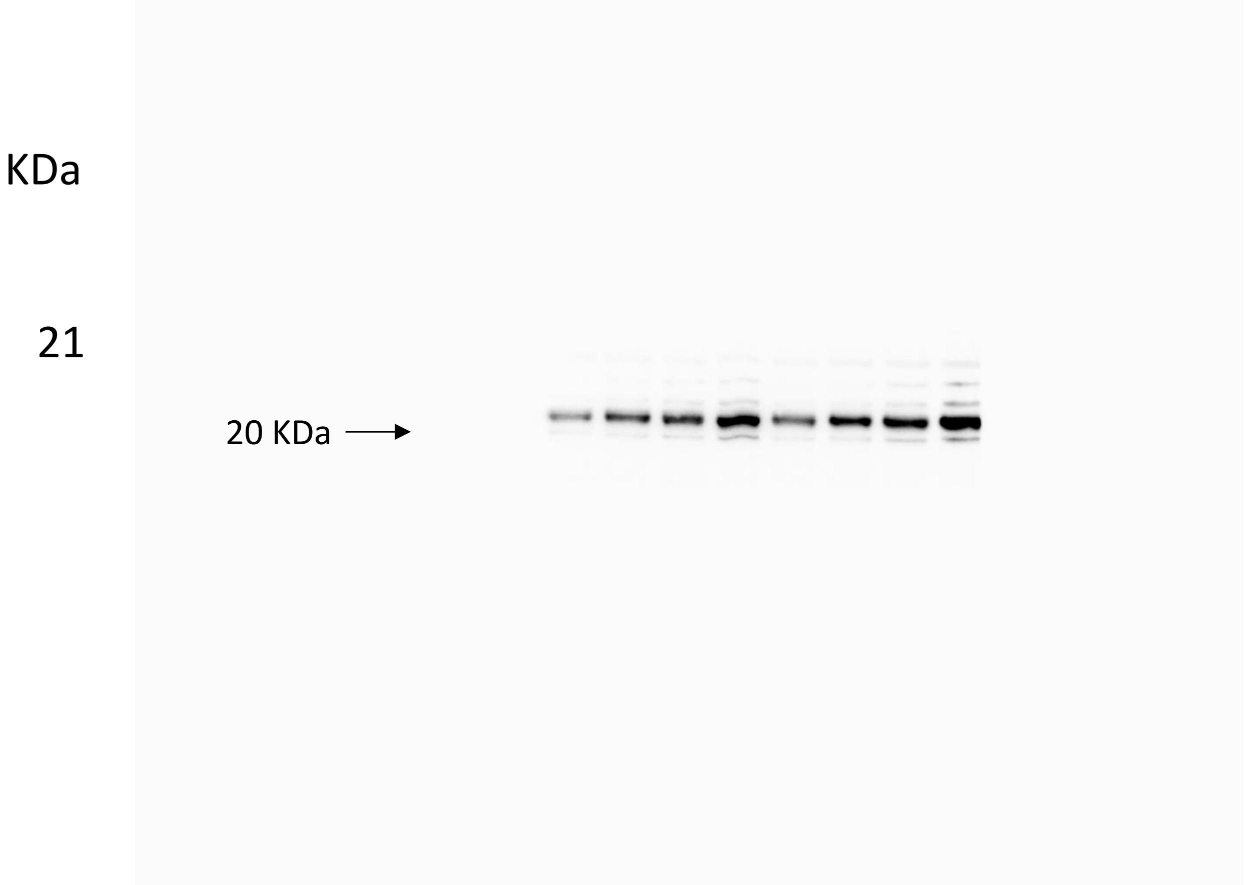

100 : 153 : 233 : 435 ; 100 : 178 : 217 : 491

Figure 5-G

Bcl-2
SH-SY5Y Cell

Control | 0.64µM BI-D1870 | 1.28µM BI-D1870 | 2.56µM BI-D1870 ; Control | 0.64µM BI-D1870 | 1.28µM BI-D1870 | 2.56µM BI-D1870

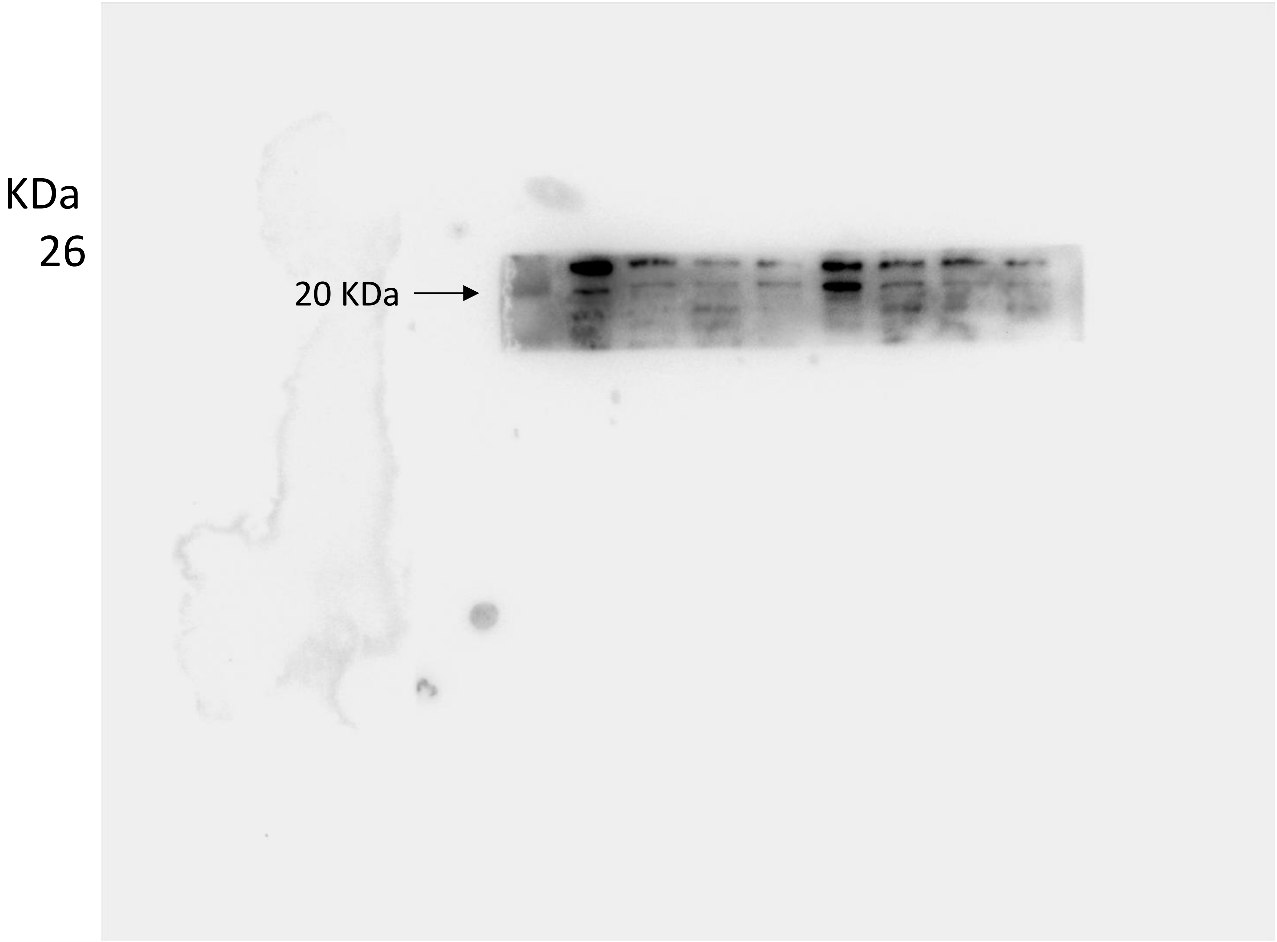

100 : 63.5 :48.1 : 126.1 ; 100 : 51.1 : 44.2 : 19.4

Figure 5-G

Caepase 3
Cleaved-caspase 3
SH-SY5Y Cell

Control | 0.64μM BI-D1870 | 1.28μM BI-D1870 | 2.56μM BI-D1870 ; Control | 0.64μM BI-D1870 | 1.28μM BI-D1870 | 2.56μM BI-D1870

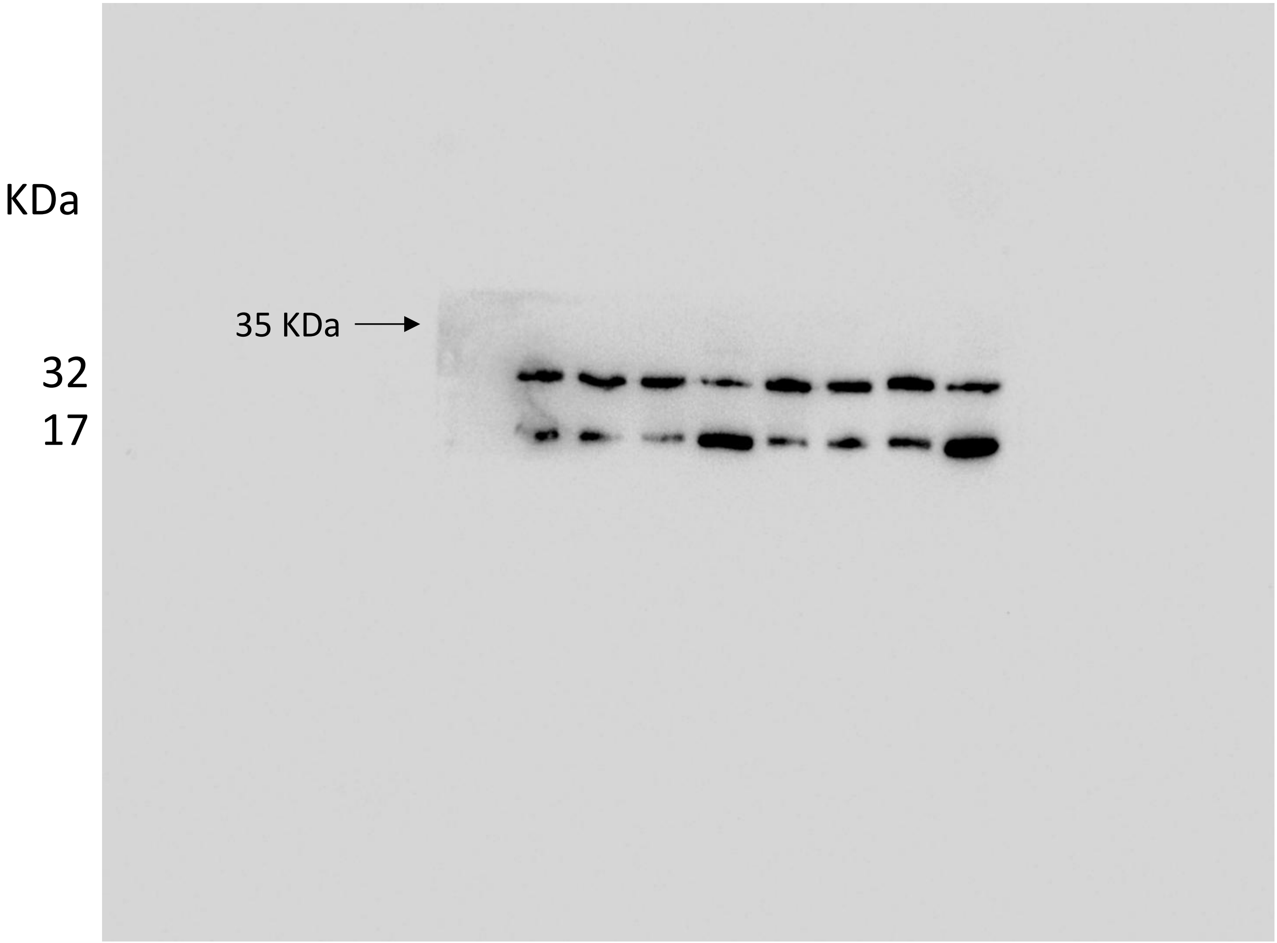

100 : 164 :198 : 375 ; 100 : 169 : 201 : 388

Figure 5-G

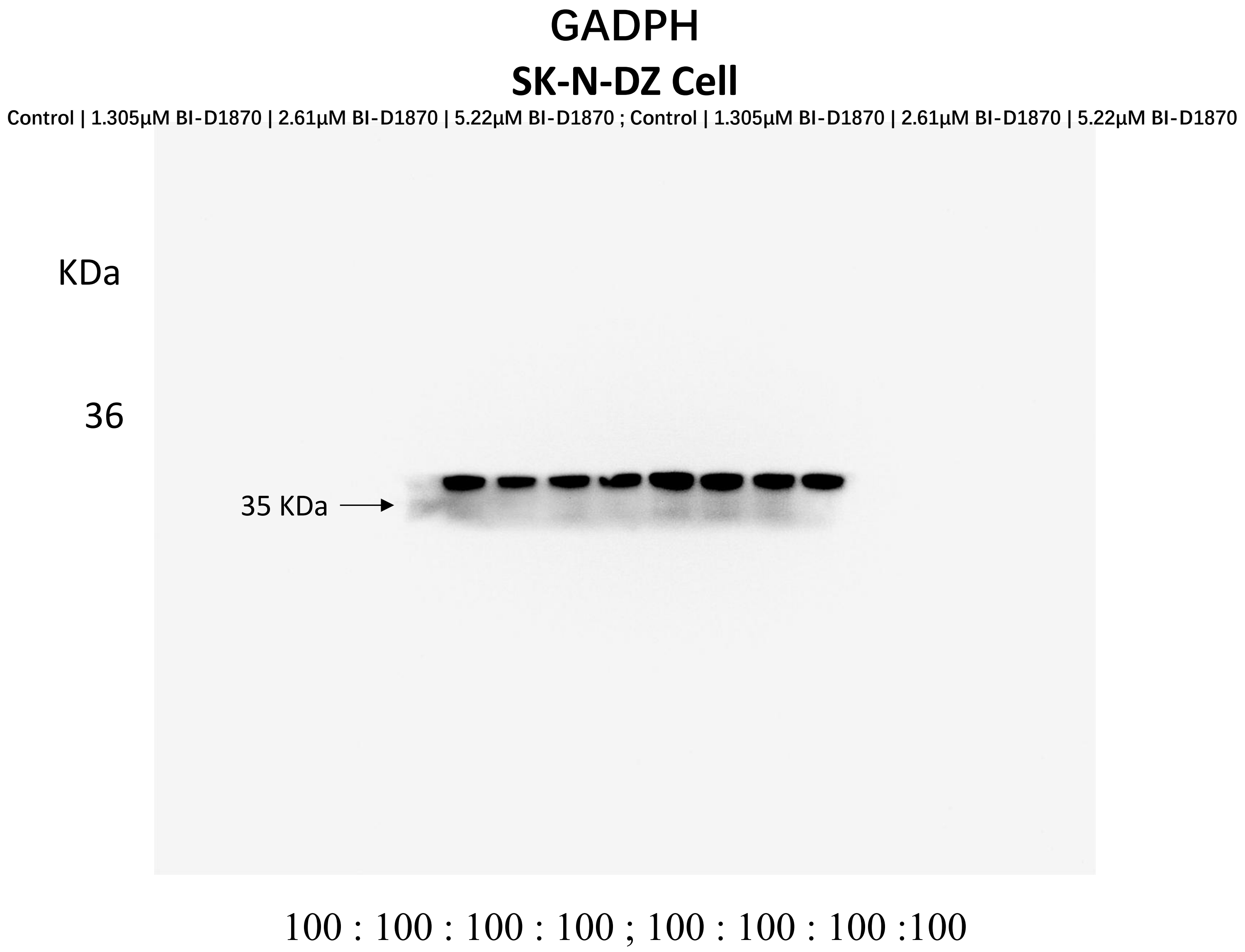
GADPH
SK-N-DZ Cell
Control | 1.305μM BI-D1870 | 2.61μM BI-D1870 | 5.22μM BI-D1870 ; Control | 1.305μM BI-D1870 | 2.61μM BI-D1870 | 5.22μM BI-D1870
KDa
36
35 KDa
100 : 100 : 100 : 100 ; 100 : 100 : 100 :100

Figure 5-H

Bax

SK-N-DZ Cell

Control | 1.305μM BI-D1870 | 2.61μM BI-D1870 | 5.22μM BI-D1870 ; Control | 1.305μM BI-D1870 | 2.61μM BI-D1870 | 5.22μM BI-D1870

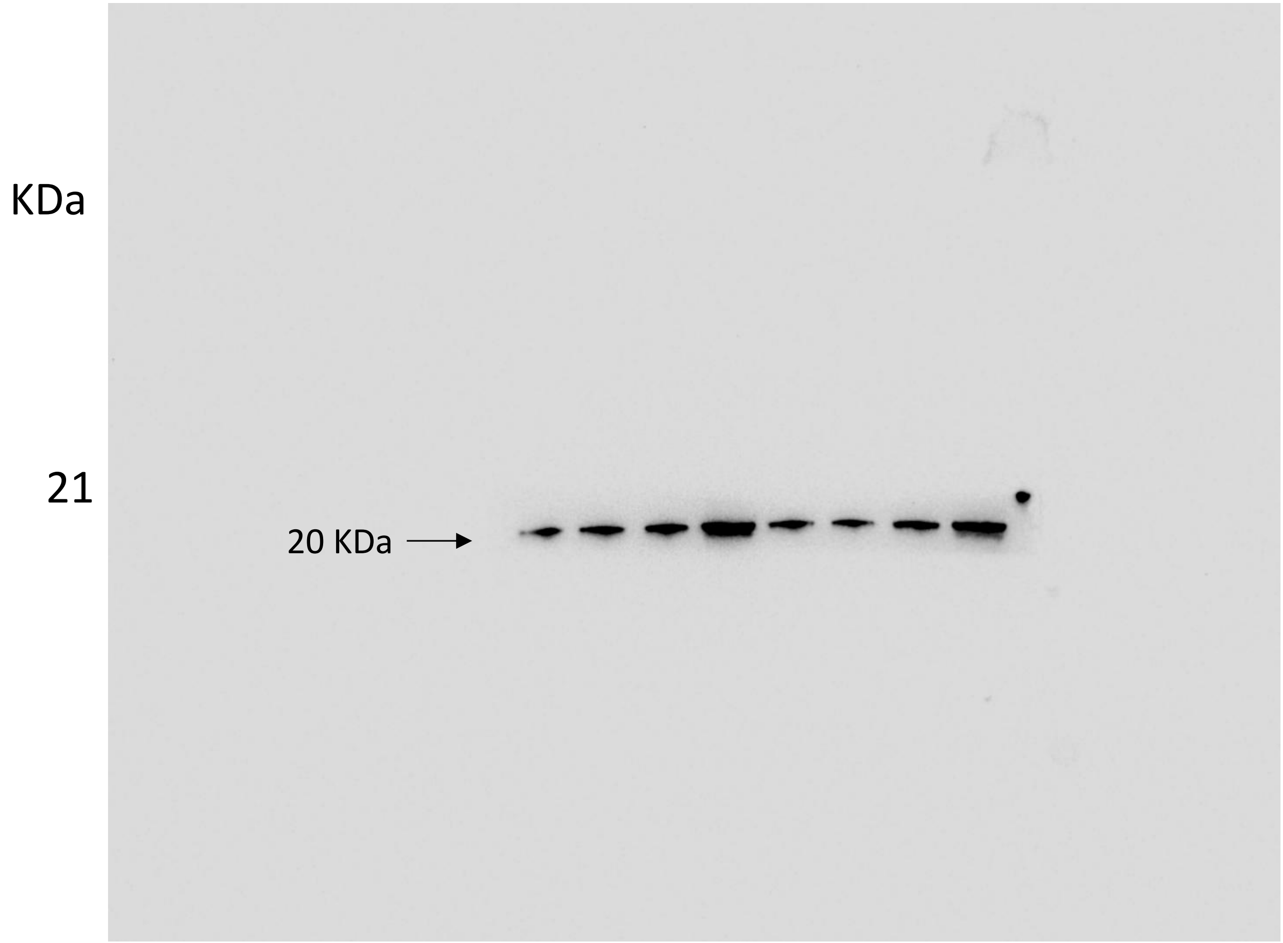

100 : 124 :160 : 308 ; 100 : 122 : 172 : 311

Figure 5-H

Bcl-2

SK-N-DZ Cell

Control | 1.305μM BI-D1870 | 2.61μM BI-D1870 | 5.22μM BI-D1870 ; Control | 1.305μM BI-D1870 | 2.61μM BI-D1870 | 5.22μM BI-D1870

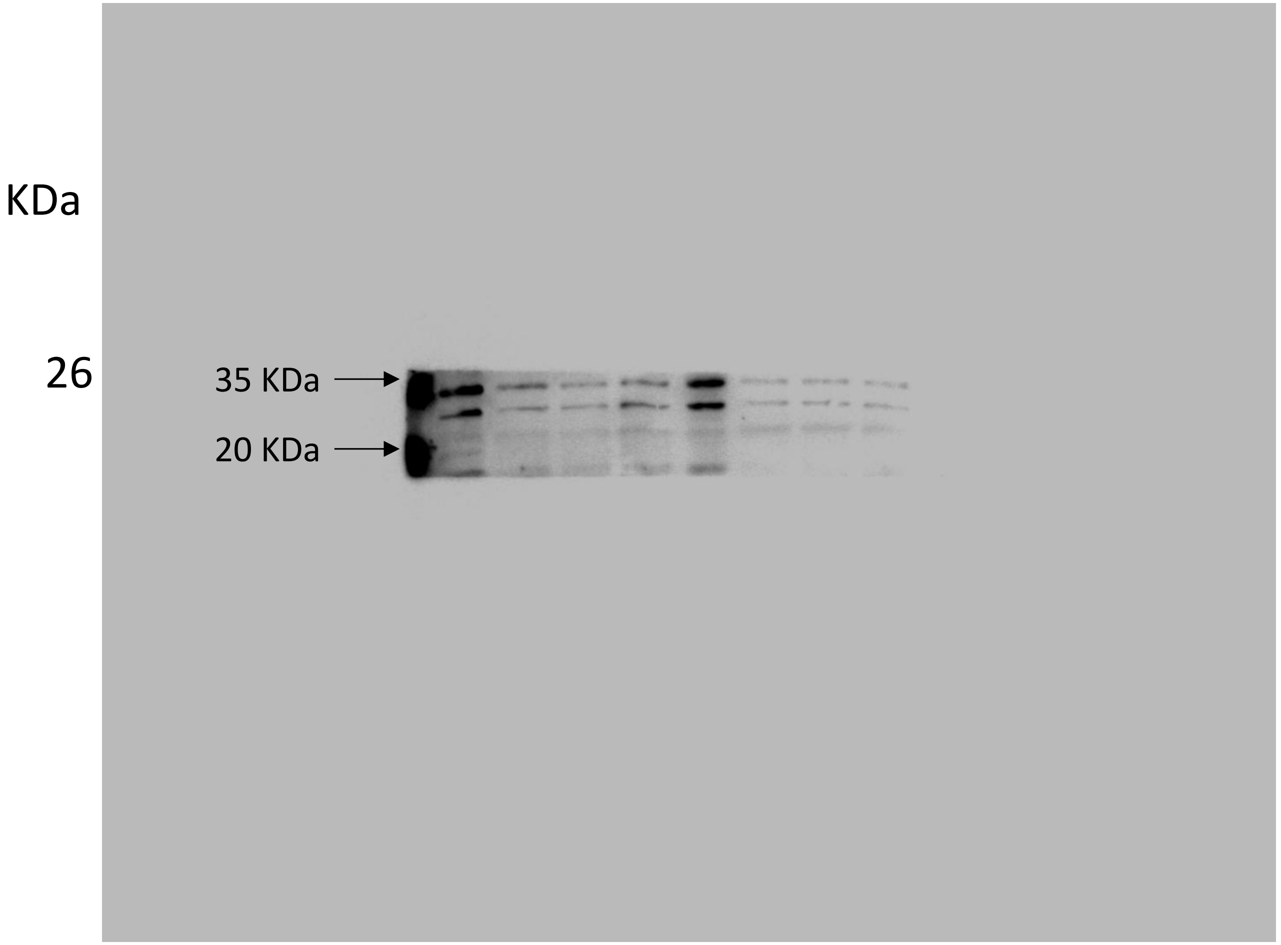

100 : 38.9 : 24.3 : 14.3 ; 100 : 33.1 : 20.1 : 12.6

Figure 5-H

Caepase 3

Cleaved-caspase 3

SK-N-DZ Cell

Control | 1.305μM BI-D1870 | 2.61μM BI-D1870 | 5.22μM BI-D1870 ; Control | 1.305μM BI-D1870 | 2.61μM BI-D1870 | 5.22μM BI-D1870

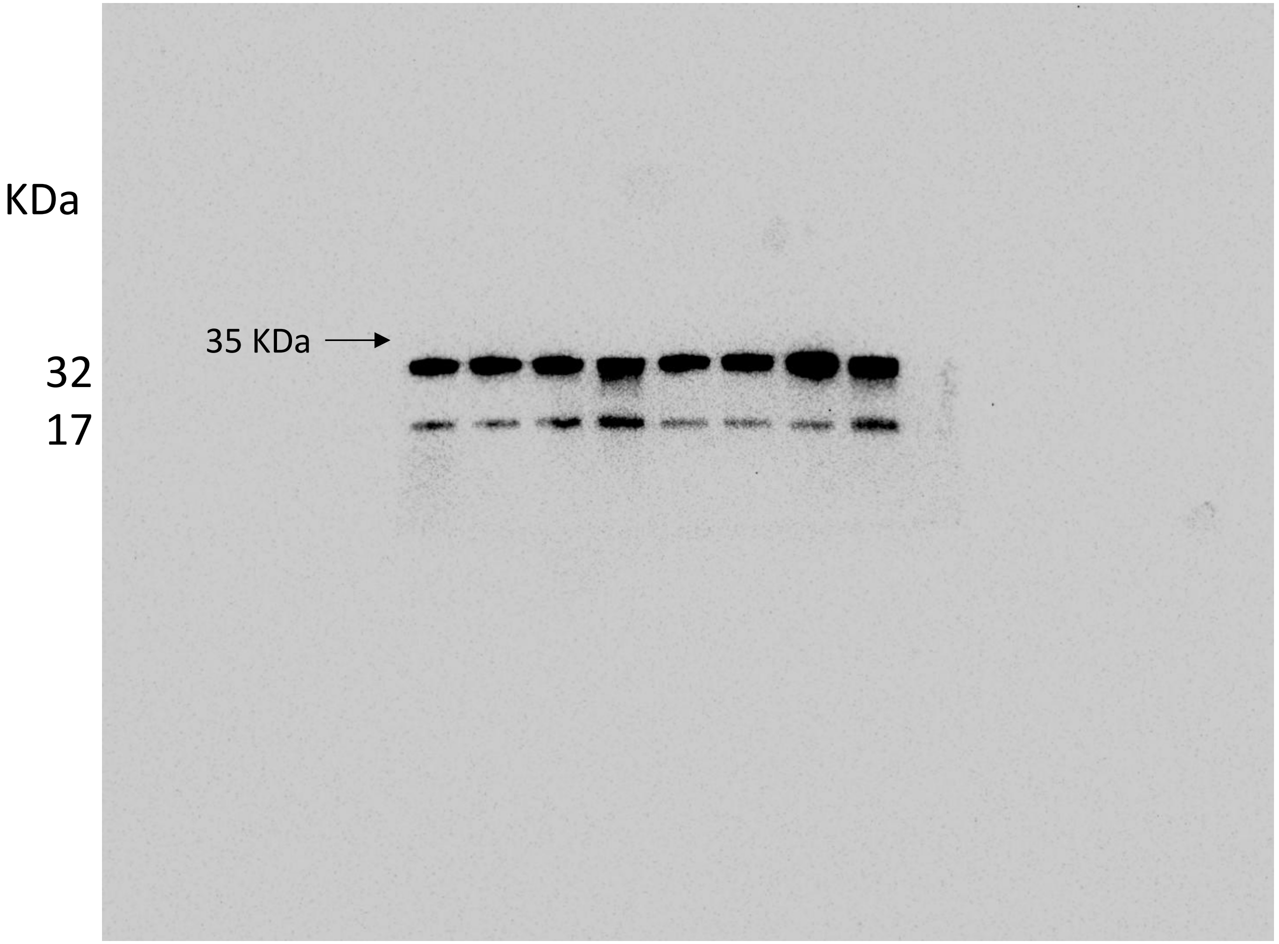

100 : 118 : 131 : 203 ; 100 : 120 : 144 : 211

Figure 5-H

GADPH

SH-SY5Y Cell

Control | 0.64μM BI-D1870 | 1.28μM BI-D1870 | 2.56μM BI-D1870

KDa

36

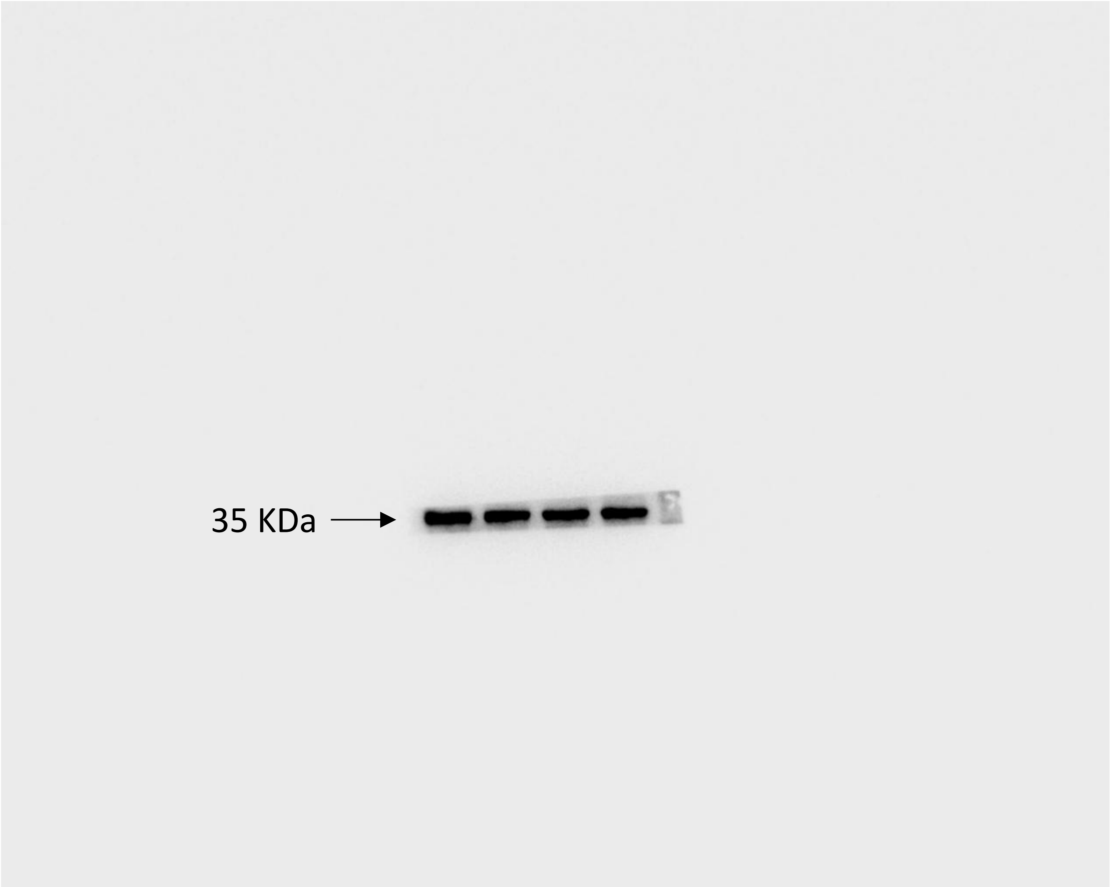

100 : 100 : 100 : 100

Figure 7-G

PI3K

SH-SY5Y Cell

Control | 0.64μM BI-D1870 | 1.28μM BI-D1870 | 2.56μM BI-D1870

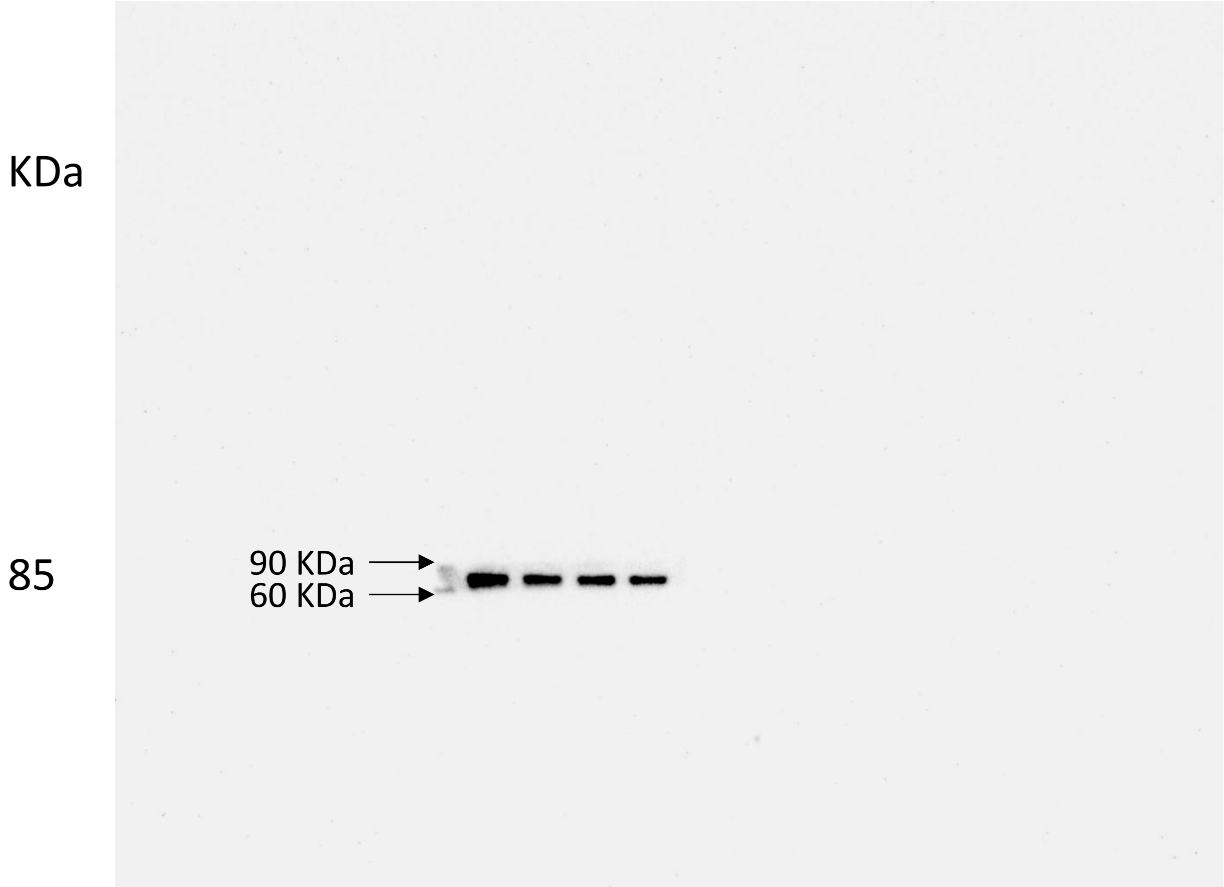

100 : 91: 99 : 92

Figure 7-G

p-PI3K

SH-SY5Y Cell

Control | 0.64μM BI-D1870 | 1.28μM BI-D1870 | 2.56μM BI-D1870

KDa

85

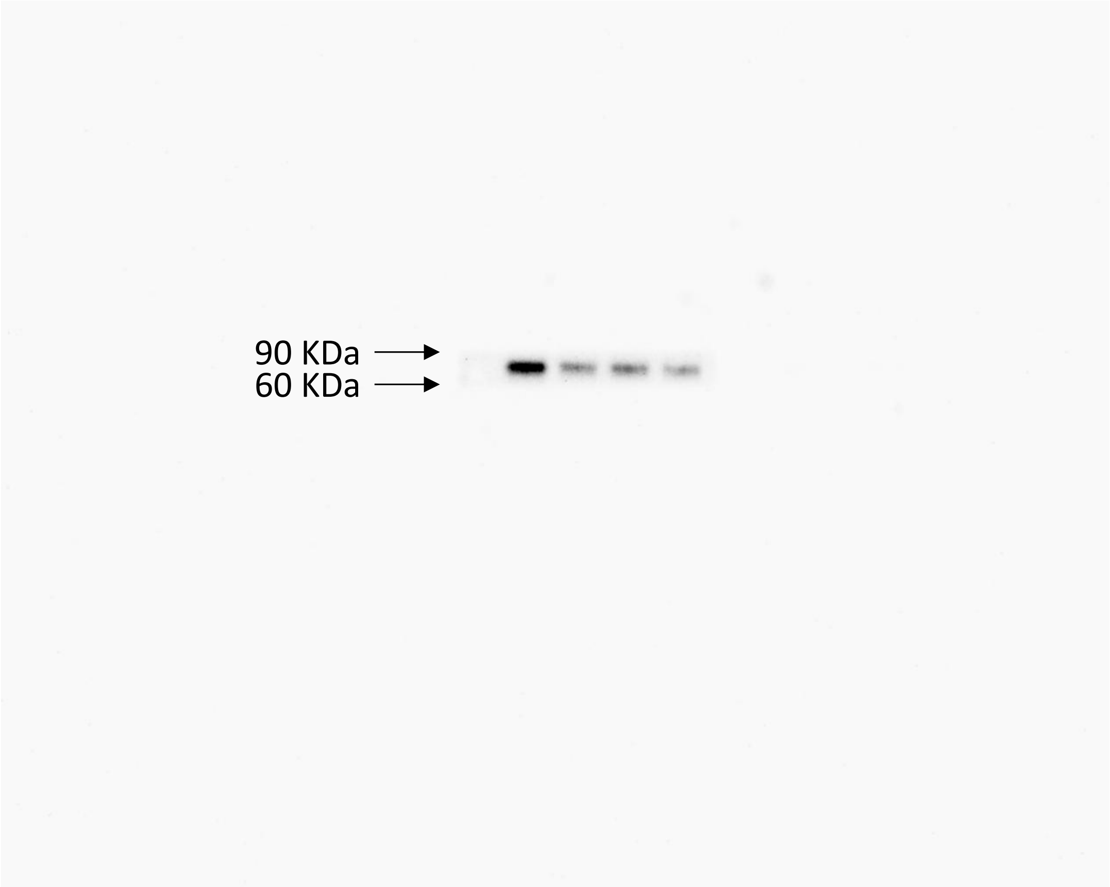

100 : 59.2 : 54.3 : 39.2

Figure 7-G

Akt

SH-SY5Y Cell

Control | 0.64μM BI-D1870 | 1.28μM BI-D1870 | 2.56μM BI-D1870

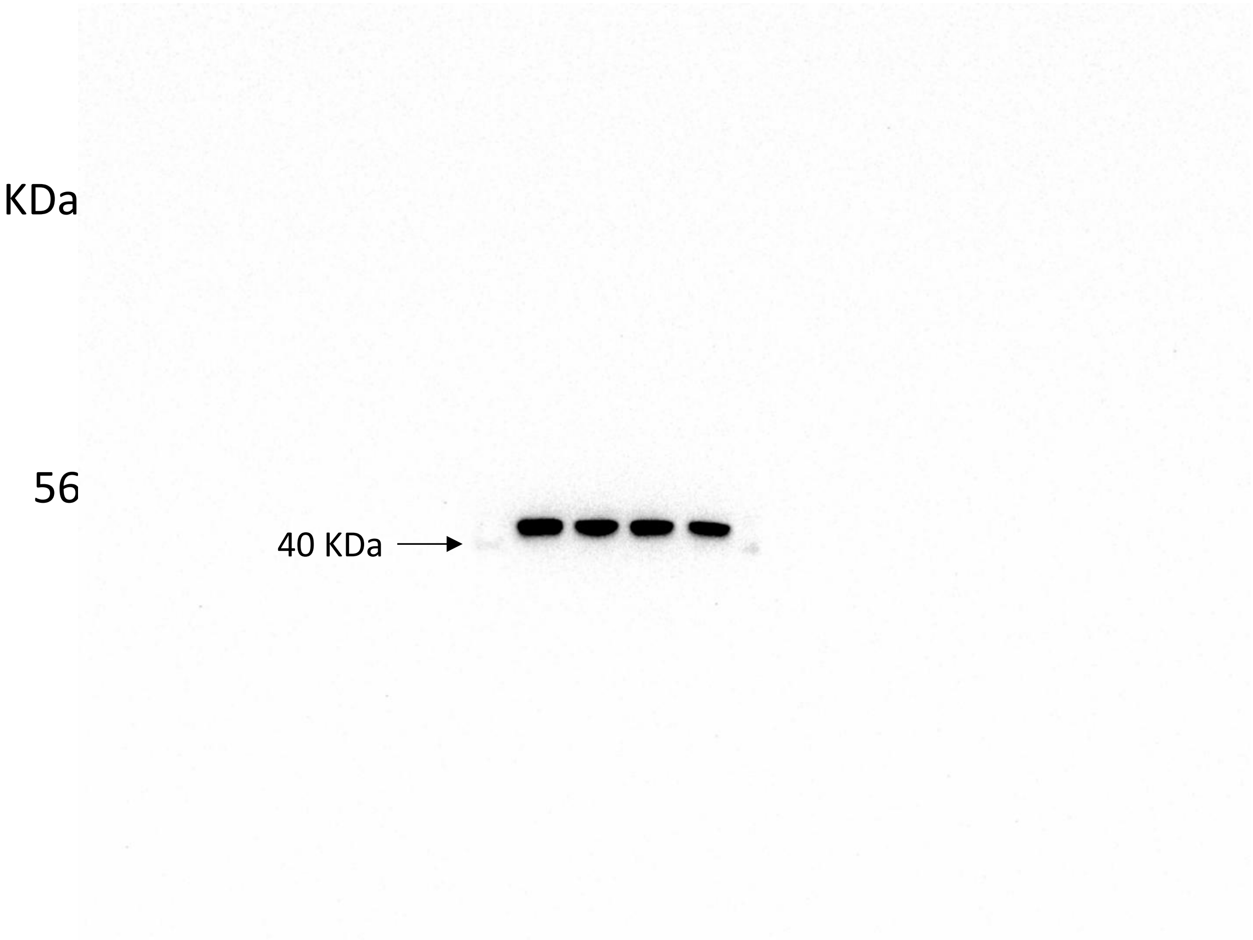

100 : 99.1 : 112 : 98.3

Figure 7-G

p-Akt

SH-SY5Y Cell

Control | 0.64μM BI-D1870 | 1.28μM BI-D1870 | 2.56μM BI-D1870

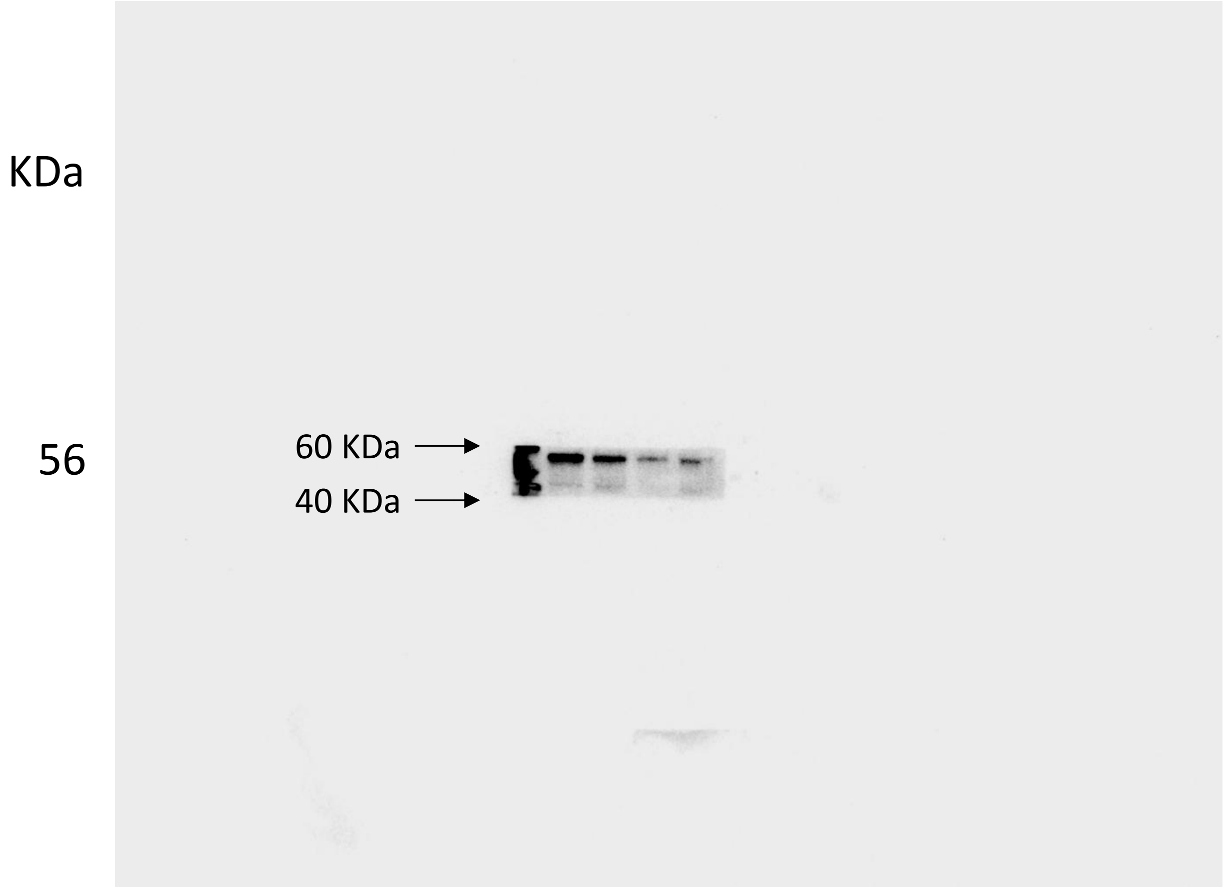

100 : 59.6 : 30.9 : 19.3

Figure 7-G

GADPH

SH-SY5Y Cell

Control | 0.64μM BI-D1870 | 1.28μM BI-D1870 | 2.56μM BI-D1870 ; Control | 0.64μM BI-D1870 | 1.28μM BI-D1870 | 2.56μM BI-D1870

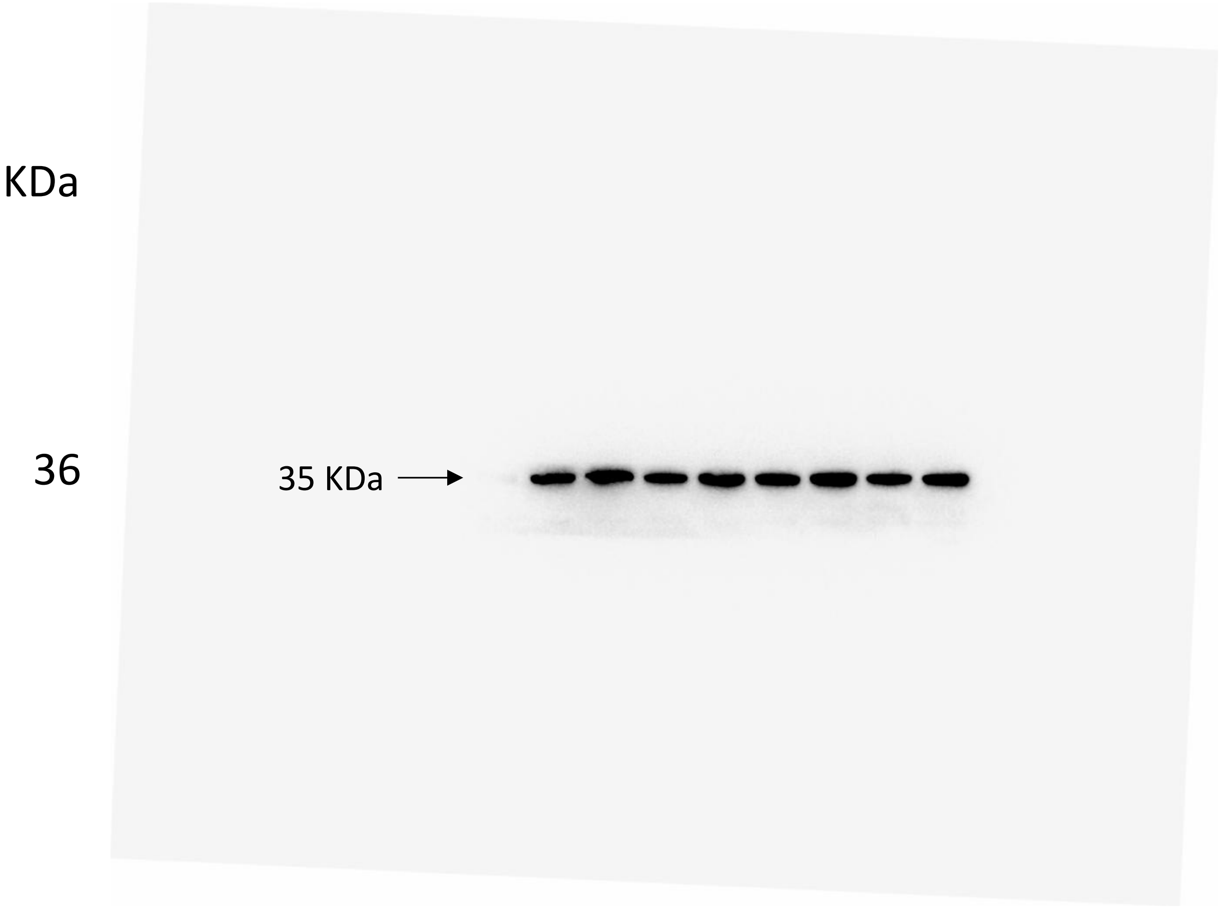

100:100:100:100 ; 100:100:100:100

Figure 7-G

RSK1
SH-SY5Y Cell

Control | 0.64μM BI-D1870 | 1.28μM BI-D1870 | 2.56μM BI-D1870 ; Control | 0.64μM BI-D1870 | 1.28μM BI-D1870 | 2.56μM BI-D1870

KDa

90

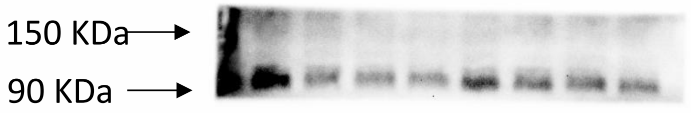

100 : 42.2: 41.7: 24.6 ; 100: 40.4 : 29.5 : 19.4

Figure 7-G

mTOR

SH-SY5Y Cell

Control | 0.64μM BI-D1870 | 1.28μM BI-D1870 | 2.56μM BI-D1870 ; Control | 0.64μM BI-D1870 | 1.28μM BI-D1870 | 2.56μM BI-D1870

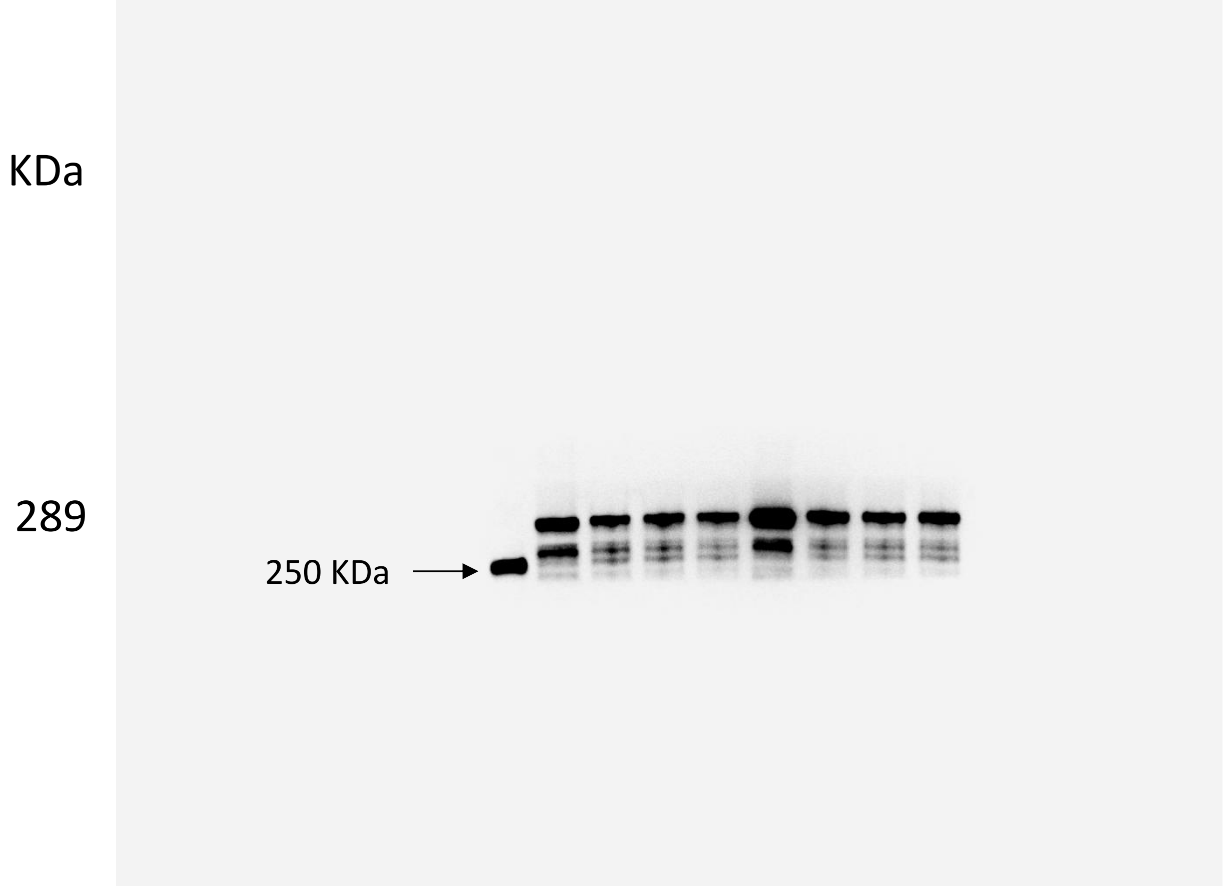

100 : 38.0 : 41.3 : 35.7 ; 100 : 62.6 : 43.3 : 37.5

Figure 7-G

p-mTOR
SH-SY5Y Cell

Control | 0.64μM BI-D1870 | 1.28μM BI-D1870 | 2.56μM BI-D1870 ; Control | 0.64μM BI-D1870 | 1.28μM BI-D1870 | 2.56μM BI-D1870

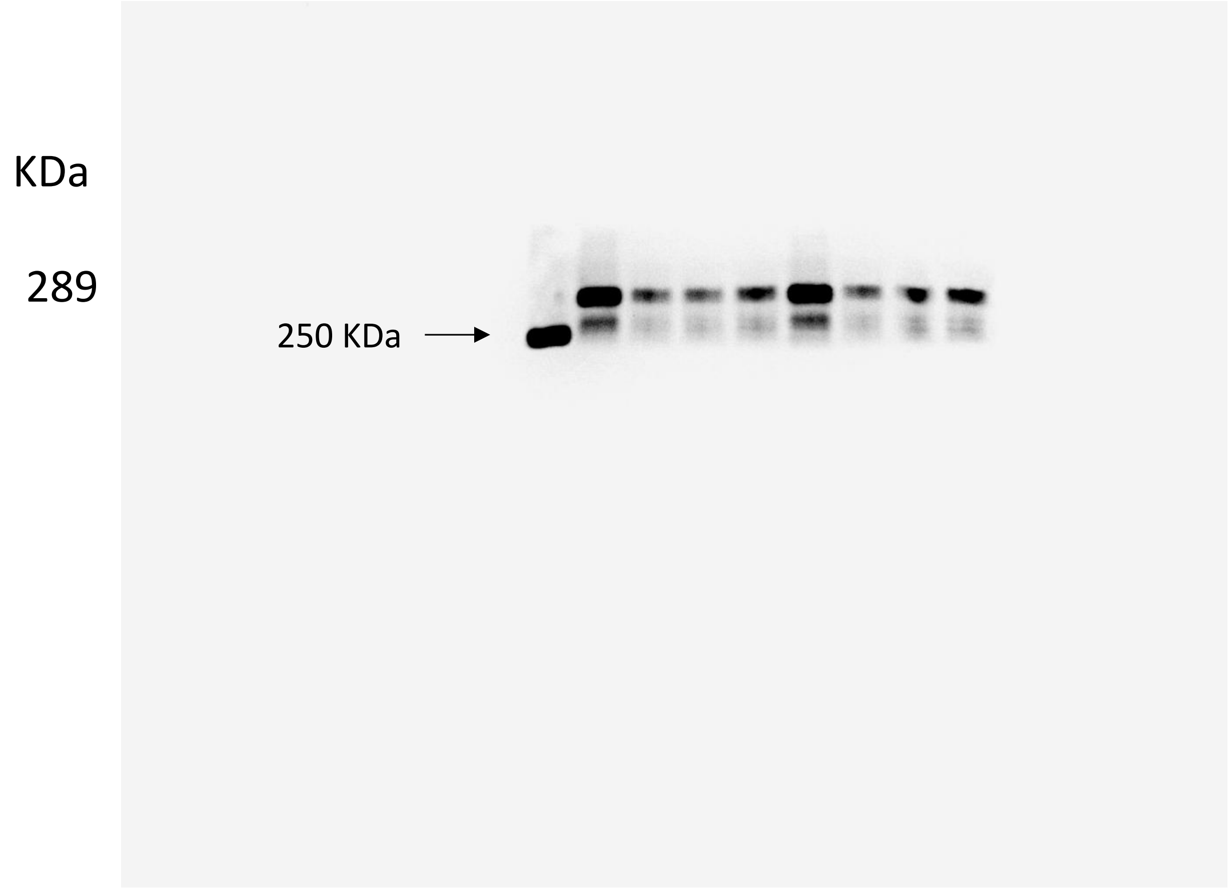

100 : 14.3 : 12.9: 17.3 ; 100 : 13.0 : 14.2 : 15.9

Figure 7-G

Raptor
SH-SY5Y Cell

Control | 0.64μM BI-D1870 | 1.28μM BI-D1870 | 2.56μM BI-D1870 ; Control | 0.64μM BI-D1870 | 1.28μM BI-D1870 | 2.56μM BI-D1870

KDa

149 150 KDa →

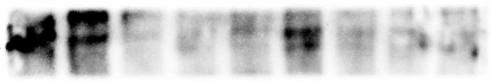

100 : 25.3 : 20.9: 16.3 ; 100 : 37.9: 37.8 : 25.8

Figure 7-G

RPS1

SH-SY5Y Cell

Control | 0.64μM BI-D1870 | 1.28μM BI-D1870 | 2.56μM BI-D1870 ; Control | 0.64μM BI-D1870 | 1.28μM BI-D1870 | 2.56μM BI-D1870

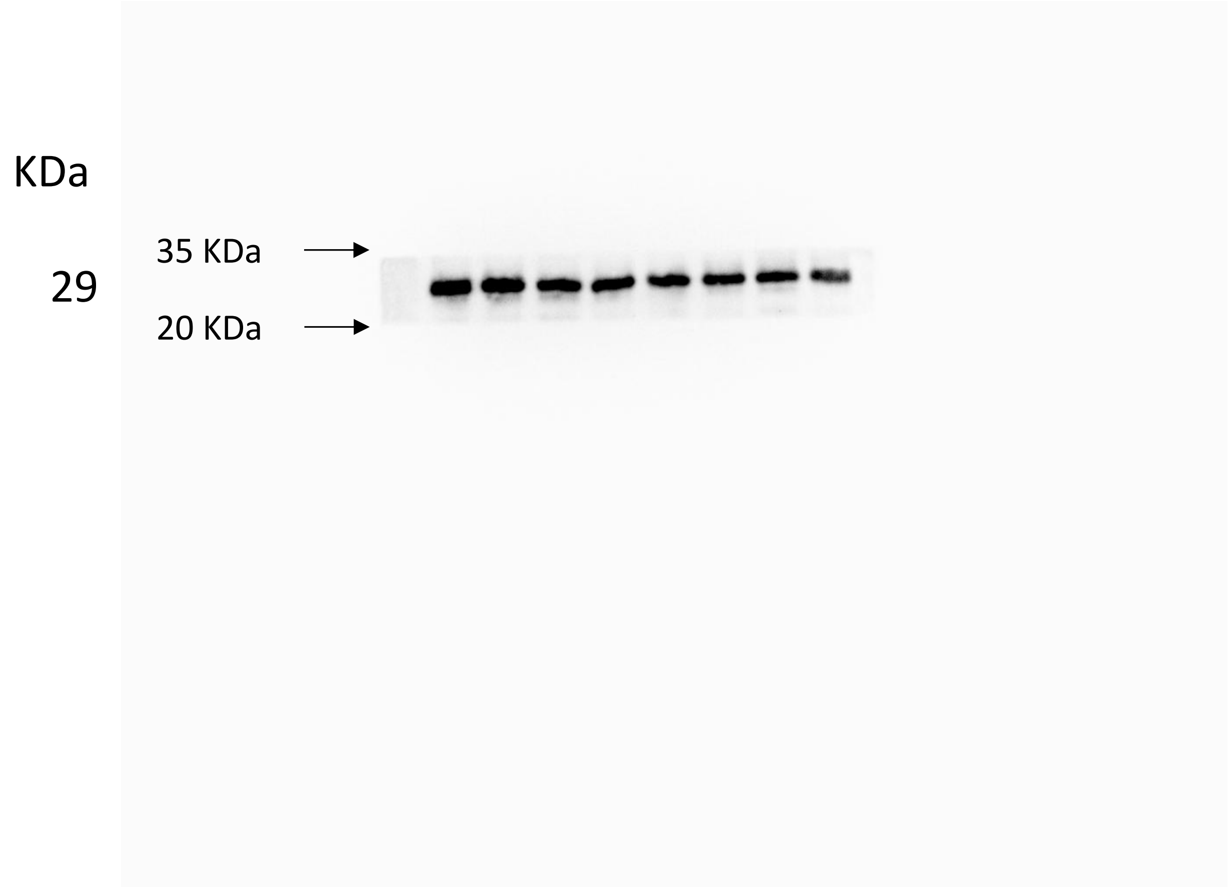

100 : 103 : 96 : 86 ; 100 : 97 : 92 : 83

Figure 7-G

p-RPS1

SH-SY5Y Cell

Control | 0.64μM BI-D1870 | 1.28μM BI-D1870 | 2.56μM BI-D1870 ; Control | 0.64μM BI-D1870 | 1.28μM BI-D1870 | 2.56μM BI-D1870

KDa

29

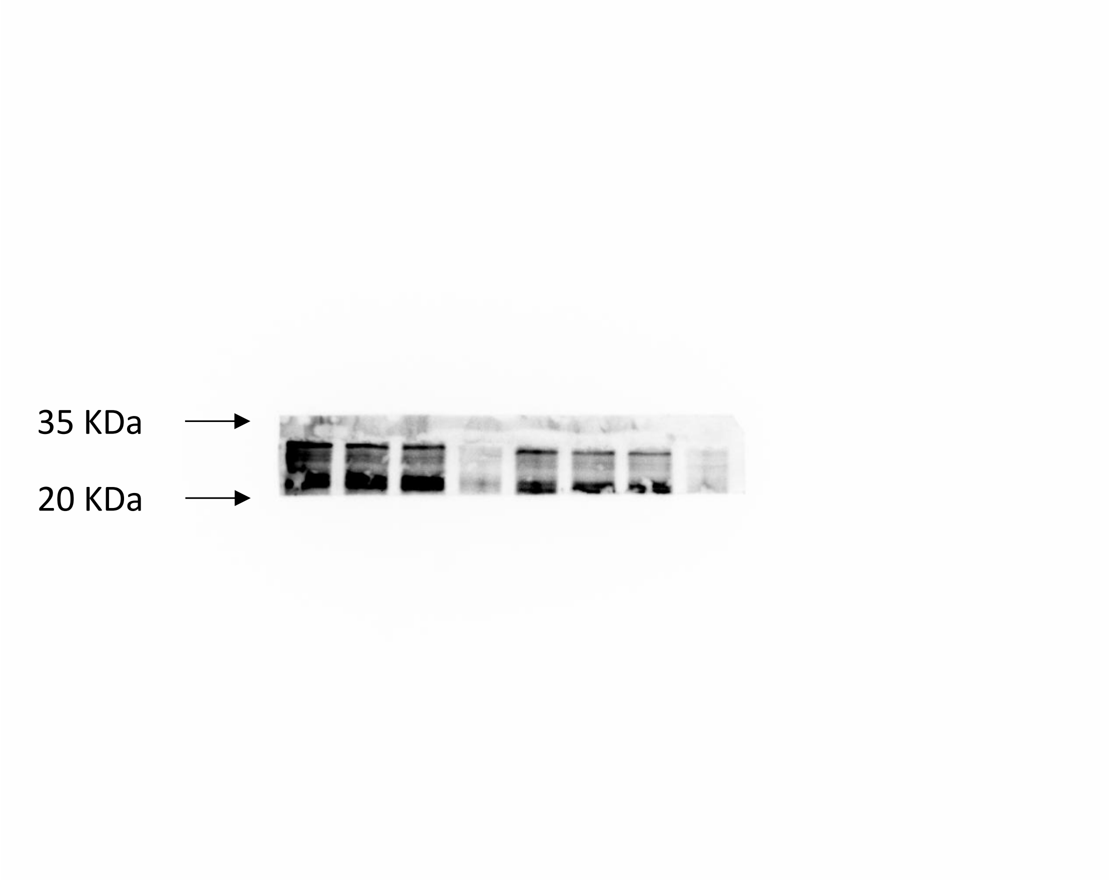

100 : 71.2 : 66.2 : 13 ; 100 : 69.5: 66.1 : 21

Figure 7-G

4E BP1
SH-SY5Y Cell

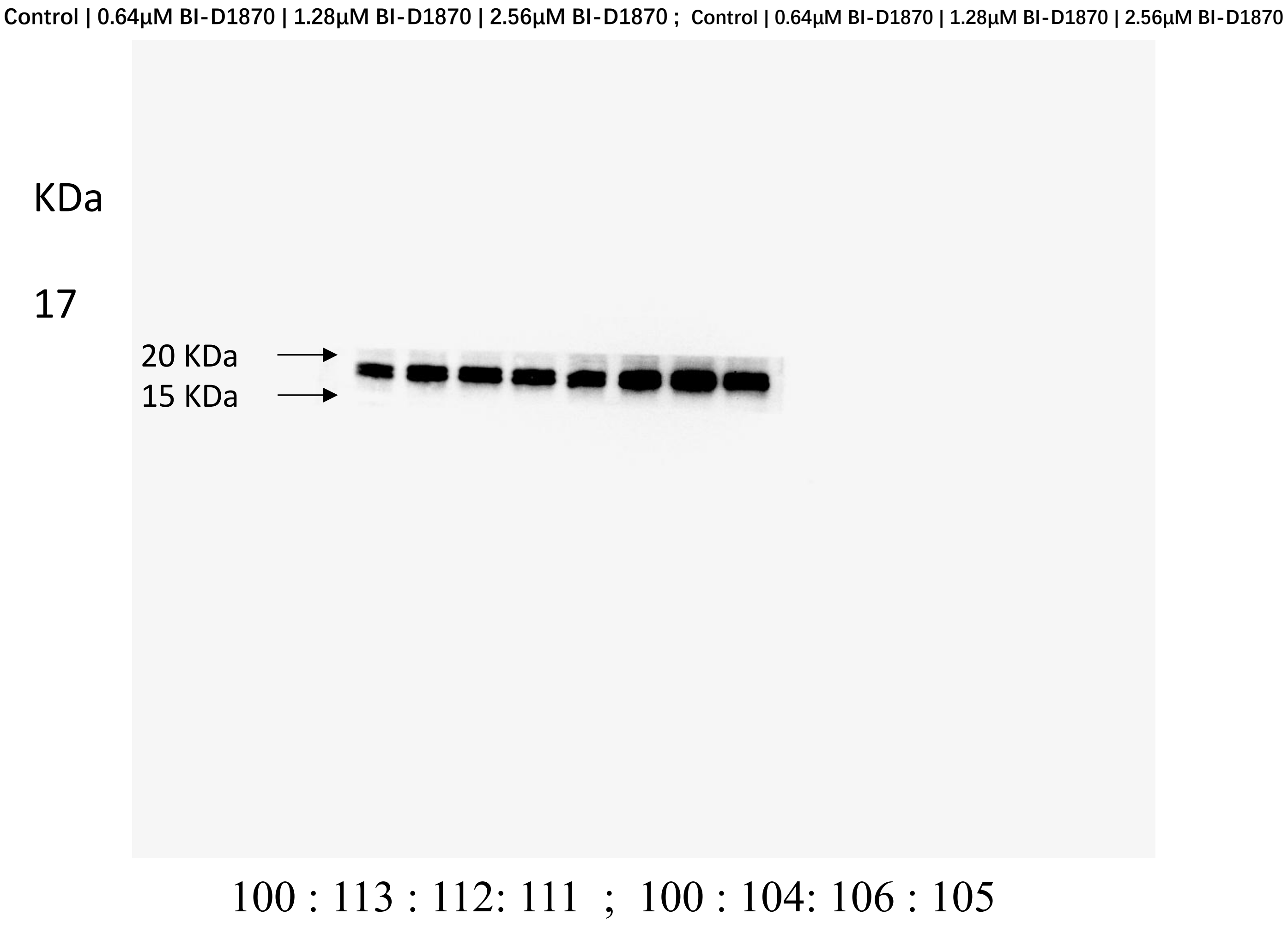

Figure 7-G

p-4E BP1

SH-SY5Y Cell

Control | 0.64μM BI-D1870 | 1.28μM BI-D1870 | 2.56μM BI-D1870 ; Control | 0.64μM BI-D1870 | 1.28μM BI-D1870 | 2.56μM BI-D1870

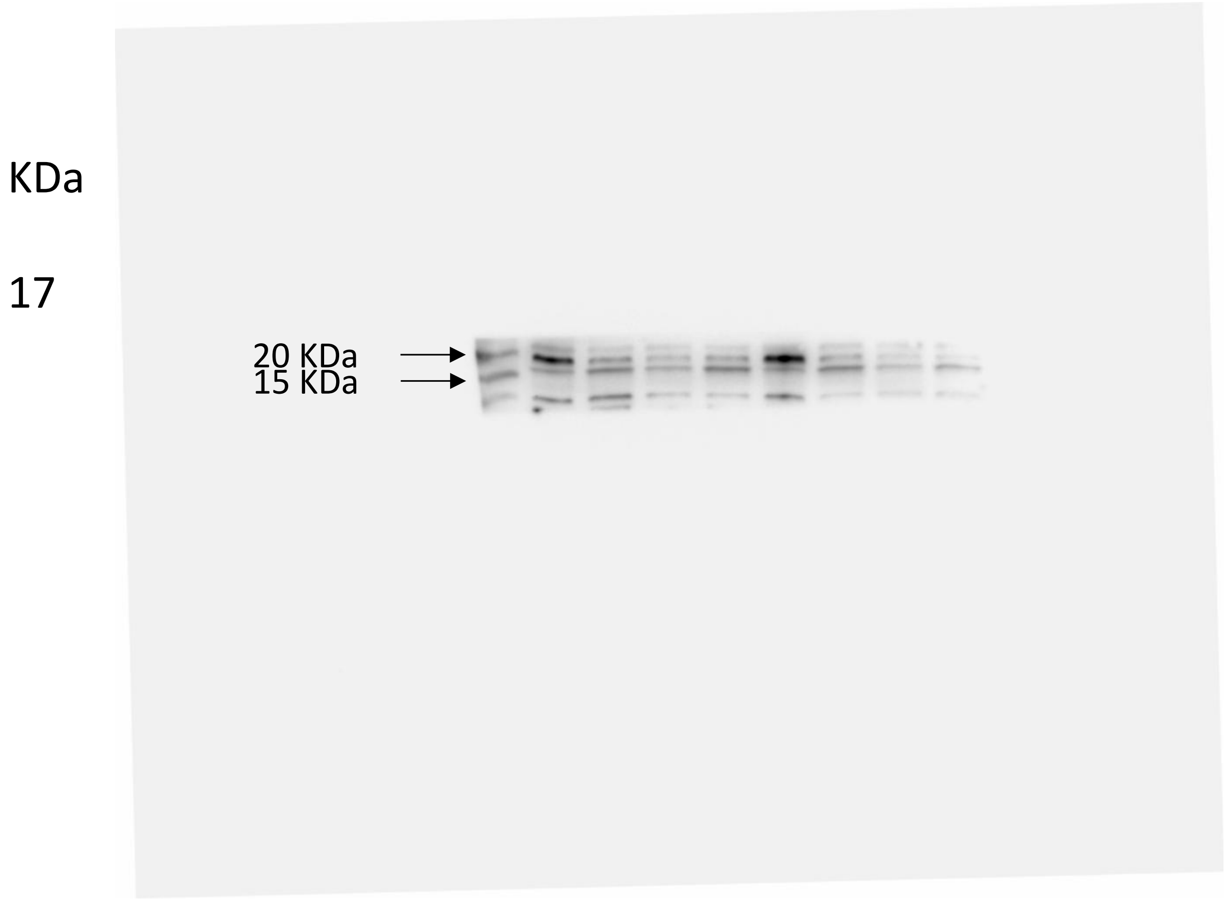

100 : 45.1 : 20.2 : 22.4 ; 100 : 27.2 : 12 : 8

Figure 7-G

CLIP170

SH-SY5Y Cell

Control | 0.64μM BI-D1870 | 1.28μM BI-D1870 | 2.56μM BI-D1870 ; Control | 0.64μM BI-D1870 | 1.28μM BI-D1870 | 2.56μM BI-D1870

KDa

170

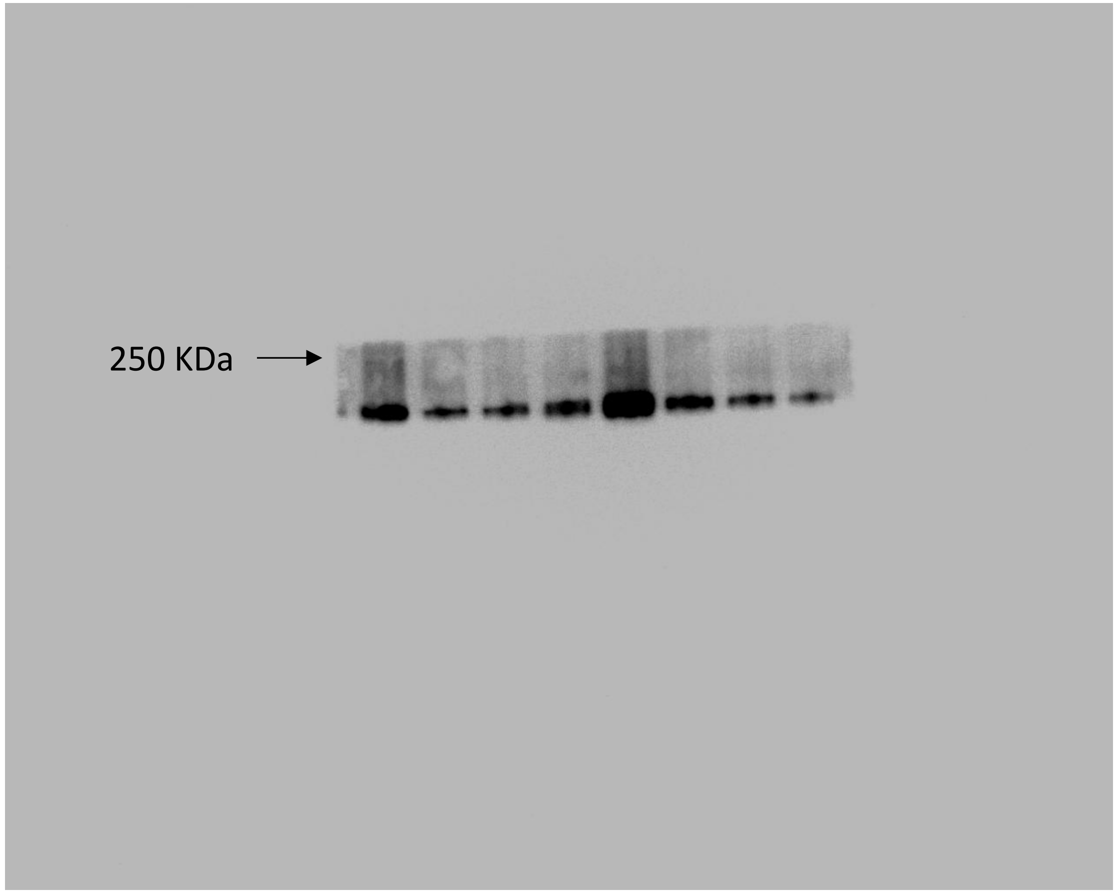

100 : 22 : 20 : 23 ; 100 : 46.3 : 25 : 7

Figure 7-G

Aurora A
SH-SY5Y Cell

MARKER | Control | 0.64μM BI-D1870 | 1.28μM BI-D1870 | 2.56μM BI-D1870 ; Control | 0.64μM BI-D1870 | 1.28μM BI-D1870 | 2.56μM BI-D1870

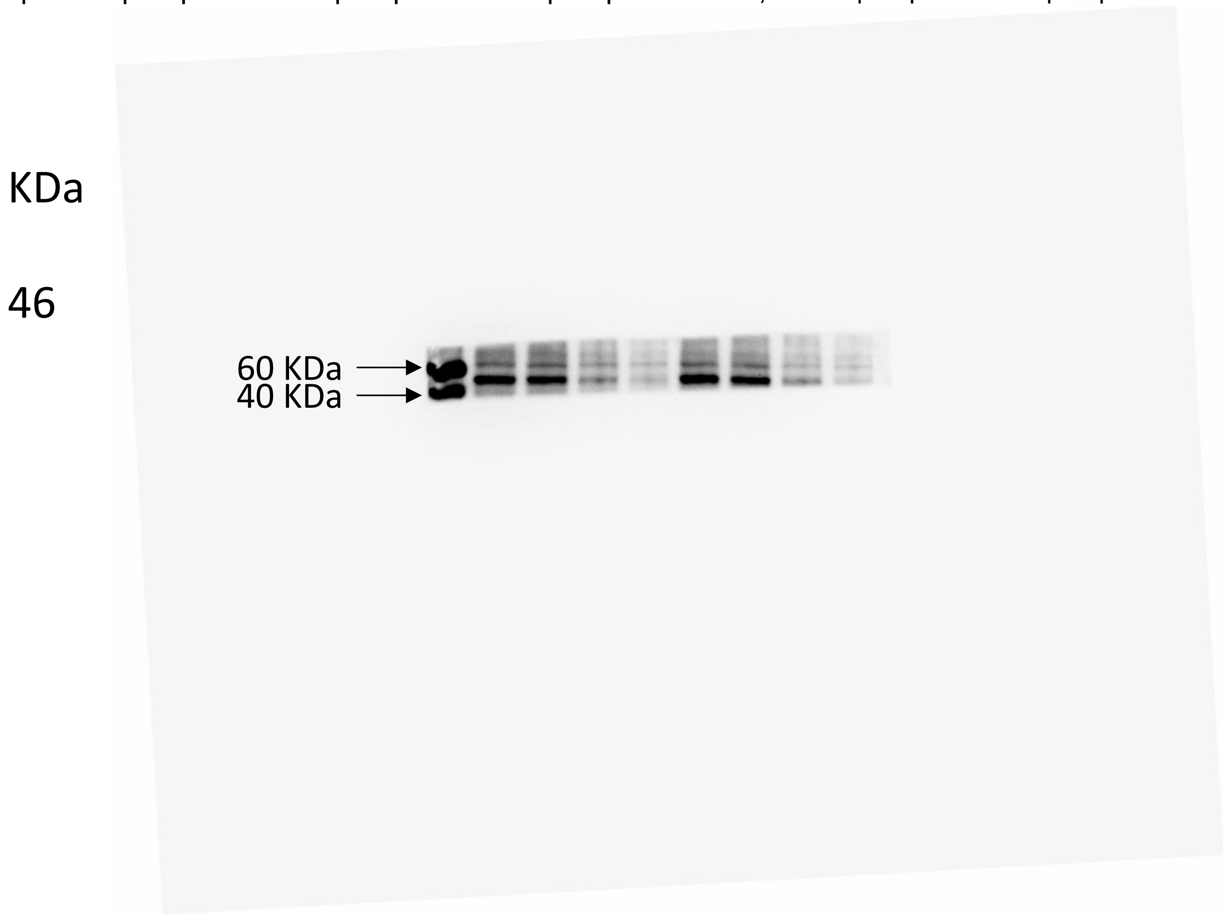

100 : 66 : 40.1 : 33 ; 100 : 57.3: 35 : 27

Figure 7-G

CDK1

SH-SY5Y Cell

Control | 0.64μM BI-D1870 | 1.28μM BI-D1870 | 2.56μM BI-D1870 ; Control | 0.64μM BI-D1870 | 1.28μM BI-D1870 | 2.56μM BI-D1870

KDa

34

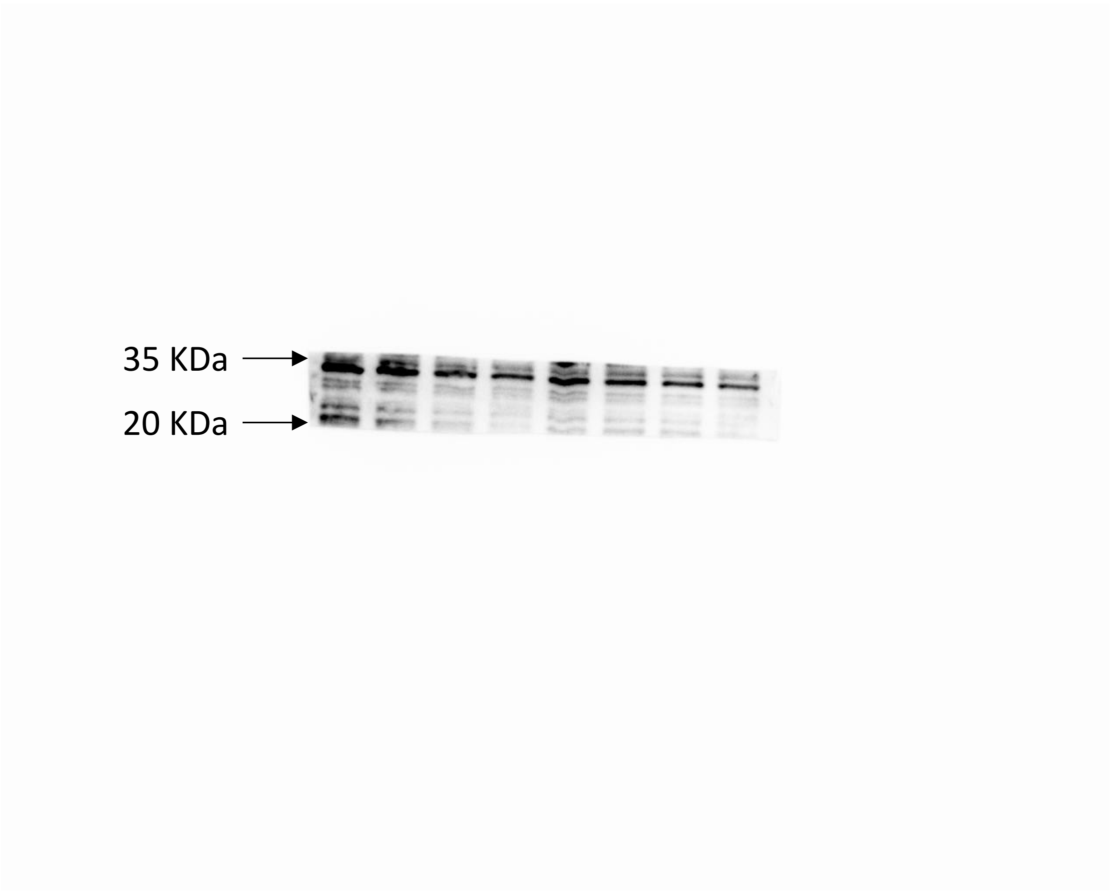

100 : 79.9 : 62.0 : 49 ; 100 : 72.7: 55.3 : 32.6

Figure 7-G

GAPDH

SH-SY5Y Cell

Control | 1.28μM BI-D1870 6h | 1.28μM BI-D1870 12h

KDa

36

35 KDa →

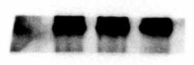

100 :100 : 100

Figure 7-I

PI3K

SH-SY5Y Cell

Control | 1.28μM BI-D1870 6h | 1.28μM BI-D1870 12h

KDa

85

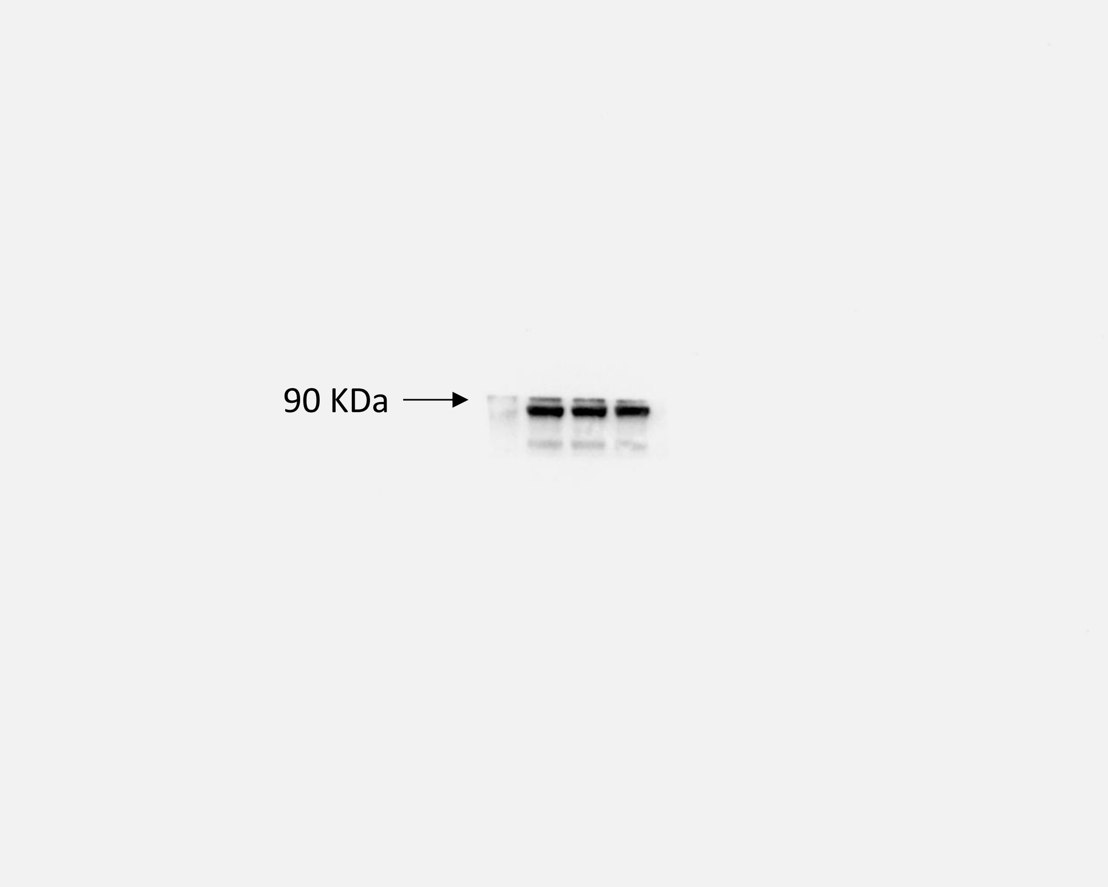

100 :102 : 100.5

Figure 7-I

p-PI3K

SH-SY5Y Cell

Control | 1.28μM BI-D1870 6h | 1.28μM BI-D1870 12h

KDa

85

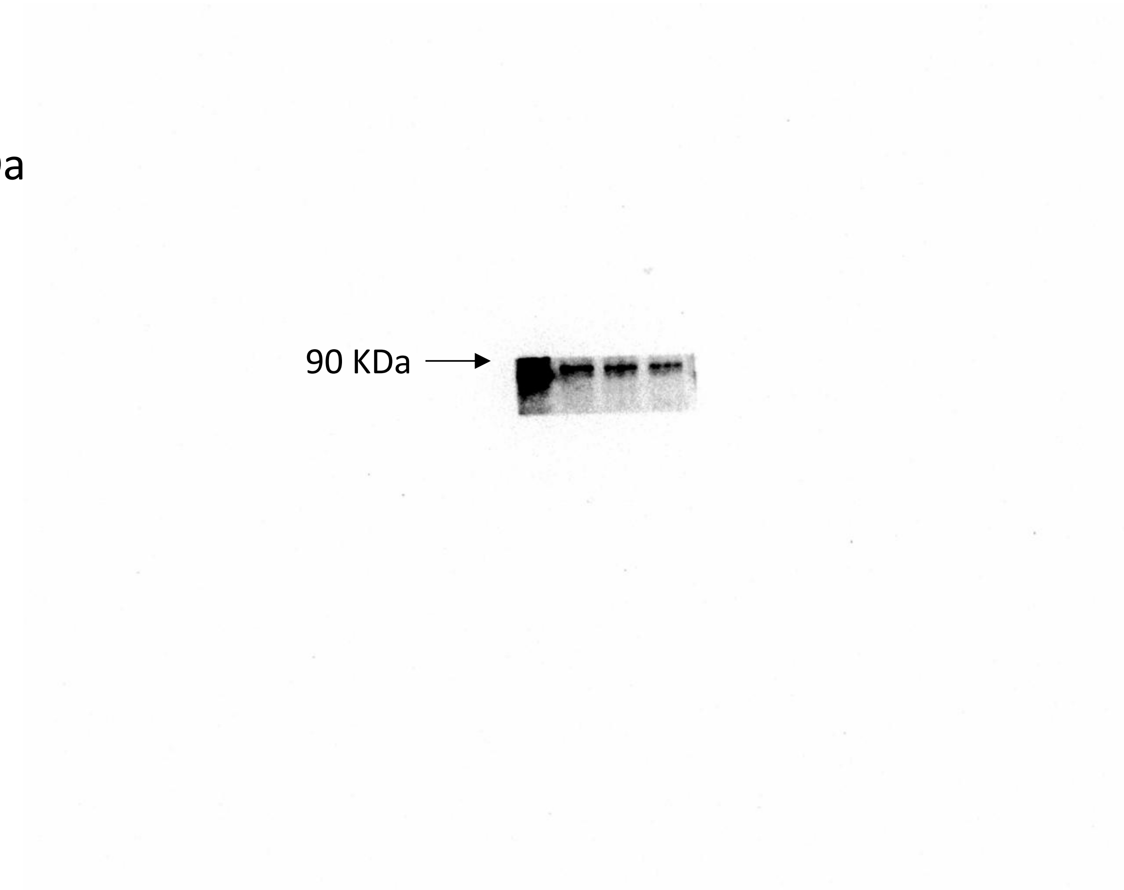

100 : 95.5 : 53.6

Figure 7-I

Akt

SH-SY5Y Cell

Control | 1.28μM BI-D1870 6h | 1.28μM BI-D1870 12h

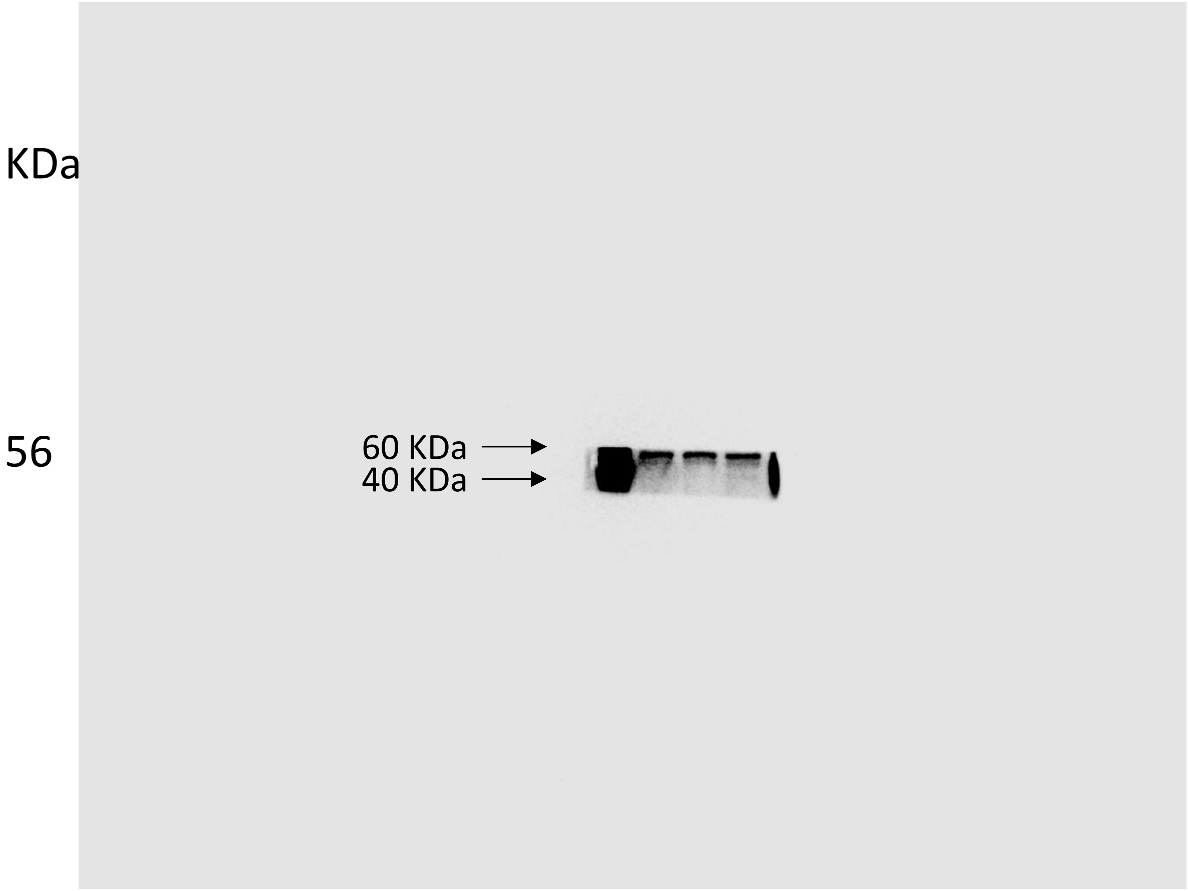

100 : 99.5 : 98.6

Figure 7-I

p-Akt

SH-SY5Y Cell

Control | 1.28μM BI-D1870 6h | 1.28μM BI-D1870 12h

KDa

56

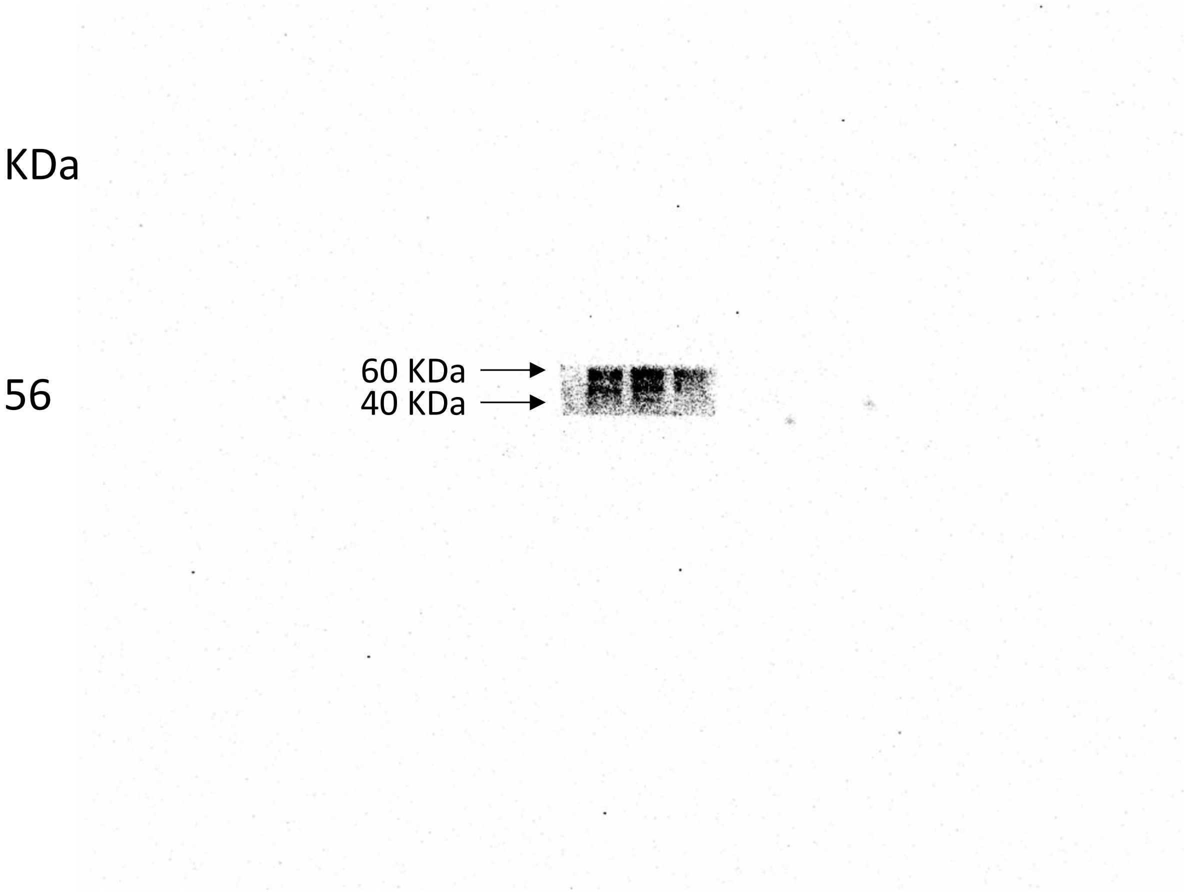

100 : 102.1 : 71.3

Figure 7-I
